# Designer Adaptor Proteins for Functional Conversion of Peptides to Small-Molecule Ligands toward In-Cell Catalytic Protein Modification

**DOI:** 10.1021/acscentsci.3c00930

**Published:** 2023-10-25

**Authors:** Akiko Fujimura, Hisashi Ishida, Tamiko Nozaki, Shuhei Terada, Yuto Azumaya, Tadashi Ishiguro, Yugo R. Kamimura, Tomoya Kujirai, Hitoshi Kurumizaka, Hidetoshi Kono, Kenzo Yamatsugu, Shigehiro A. Kawashima, Motomu Kanai

**Affiliations:** † Graduate School of Pharmaceutical Sciences, The University of Tokyo, Tokyo 113-0033, Japan; ‡ Institute for Quantum Life Science, National Institutes for Quantum Science and Technology, Chiba 263-8555, Japan; § Institute for Quantitative Biosciences, The University of Tokyo, Tokyo 113-0032, Japan

## Abstract

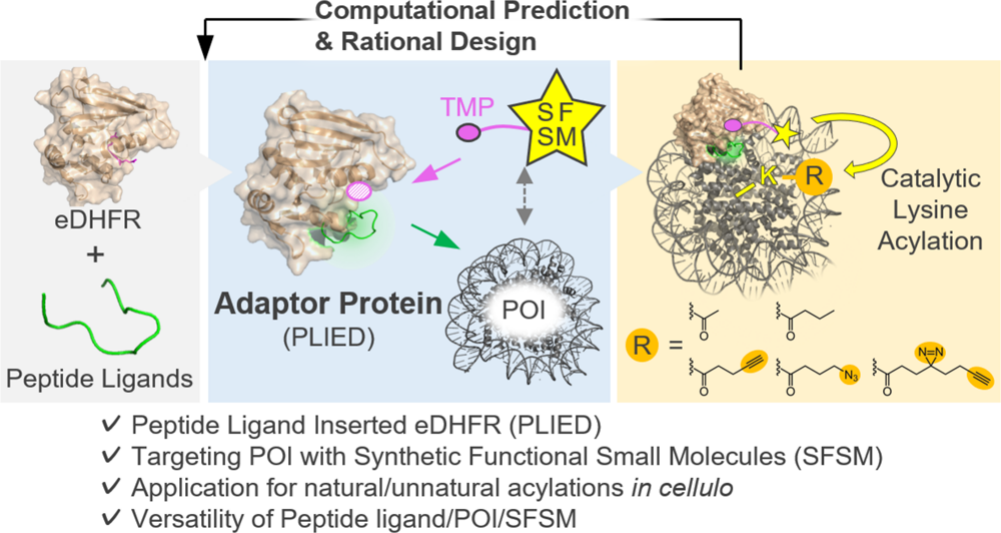

Peptides are privileged ligands for diverse biomacromolecules, including proteins; however, their utility is often limited due to low membrane permeability and in-cell instability. Here, we report peptide ligand-inserted eDHFR (PLIED) fusion protein as a universal adaptor for targeting proteins of interest (POI) with cell-permeable and stable synthetic functional small molecules (SFSM). PLIED binds to POI through the peptide moiety, properly orienting its eDHFR moiety, which then recruits trimethoprim (TMP)-conjugated SFSM to POI. Using a lysine-acylating BAHA catalyst as SFSM, we demonstrate that POI (MDM2 and chromatin histone) are post-translationally and synthetically acetylated at specific lysine residues. The residue-selectivity is predictable in an atomic resolution from molecular dynamics simulations of the POI/PLIED/TMP-BAHA (MTX was used as a TMP model) ternary complex. This designer adaptor approach universally enables functional conversion of impermeable peptide ligands to permeable small-molecule ligands, thus expanding the in-cell toolbox of chemical biology.

## Abstract 2

A peptide ligand-inserted eDHFR (PLIED) fusion protein as a universal adaptor for targeting proteins of interest (POI) with cell-permeable and stable synthetic functional small molecules.

Peptides represent a unique class of protein ligands, as the molecular size and biochemical properties of peptides are distinct from small molecules or proteins. Since the isolation and first therapeutic use of insulin in the 1920s, peptide ligands and drug candidates have been developed toward a broad range of protein targets, including extracellular hormone receptors, receptor tyrosine kinases, and intracellular proteins.^1^ Peptides can interact with broad protein surfaces that are utilized for protein–protein interactions (PPIs) in nature (Figure 1A). In addition, peptides generally furnish higher selectivity and affinity to their targets, lower toxicity, and broader structural diversity than small molecules.^2^ Peptides are also useful ligands for directing synthetic functional small molecules (SFSM) of chemical biology tools to target proteins. However, their utility has been often limited due to low cell membrane permeability and in-cell stability (Figure 1A).

**Figure 1:**
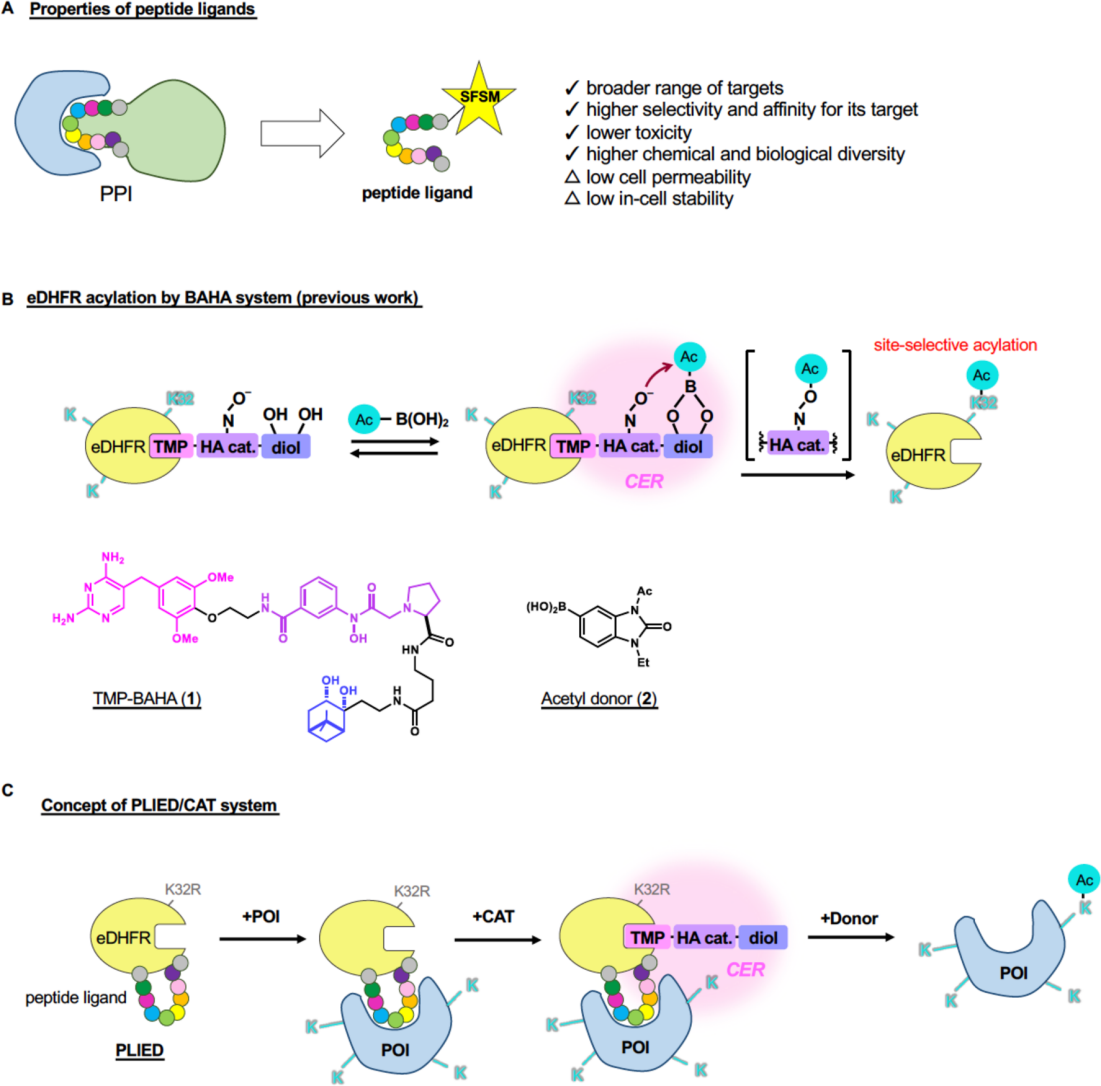
Development of PLIED/CAT system. A, Schematic representation of drawbacks of peptide ligands. B, eDHFR acylation by TMP-BAHA and acyl donor (upper). Chemical structures of TMP-BAHA **1** and acetyl donor **2** (lower). C, A strategy for protein acylation by PLIED/CAT system.

Therefore, surrogating peptides by small molecules (i.e., depeptidizing) is an important approach in drug development research. For example, peptide inhibitors prohibiting the interaction between tumor suppressor p53 and its natural antagonist murine double minute 2 (MDM2) had been developed from the wild-type p53-derived peptide by modification of each amino acid, such as the tyrosine and the tryptophan side chains.^3^ Once the pharmacophore was established from the cocrystal structure of p53/MDM2 and peptide inhibitors, small-molecule inhibitors were identified by combining structure-based design and combinatorial chemistry.^4^ However, the depeptidizing approach is not always successful. Many proteins lack a small-molecule ligand, such as chromatin histone proteins.

Chemical modification of native proteins in living cells is a fundamental process to probe, control, or manipulate protein functions. As a typical example, fluorescence labeling of a protein of interest (POI) enables spatiotemporal imaging and analysis of a target protein.^5^ For selective labeling of endogenous proteins in living cells, traceless protein labeling methods, such as ligand-directed chemistry^6^ or affinity-guided catalyst chemistry,^7^ have been developed.^8^ The proximity effect of POI–ligand recognition defines reaction efficiency as well as the target protein selectivity. Therefore, appropriate selection of the ligand is critical.

In addition to labeling POIs by a probe moiety, artificial installation of post-translational modifications (PTMs), which is physiologically catalyzed by enzymes, into a POI can modify protein functions and intervene chemical networks in living system.^9^ For example, PTMs of histone protein, such as lysine acetylation, form the eukaryotic epigenome and play an essential role in gene expression. Since dysregulation of histone PTMs is involved in various diseases such as cancer,^10^ artificial lysine acetylation of histone proteins may restore abnormal epigenome to healthy states. Toward this objective, our group has developed abiotic catalysts that can promote lysine acylation on a target protein in living cells.^11−14^ Specifically, a boronate-assisted hydroxamic acid (BAHA) catalyst^14^ recruits a boronic acid-containing acyl donor via a diol moiety, and then the reactive acyl hydroxamate intermediate is generated by the subsequent intramolecular acyl transfer to the Lewis base moiety (Figure 1B). Due to enhanced local molarity effects, the BAHA catalyst system employs micromolar acyl donor concentrations and affords minimal off-target protein reactivity. Importantly, BAHA catalyst is resistant to glutathione, a major intracellular reductant. Using affinity-guided catalysis, our group synthesized a cell-permeable and stable small-molecule catalyst, TMP-BAHA **1**, which promotes synthetic lysine acylation of *E. coli* dihydrofolate reductase (eDHFR) expressed in cultured human cells.^14^ Catalyst **1** comprises a protein ligand for eDHFR (trimethoprim: TMP), a hydroxamate Lewis base catalytic center (HA cat.), and a diol moiety (Figure 1B). Among the six lysine residues (K32, K38, K58, K76, K106, and K109) in eDHFR, **1** combined with the boronic acid-containing acyl donor **2** exclusively acylates K32, which is proximal to the TMP-binding pocket, indicating that K32 is the only accessible lysine residue from the catalytic center of **1** when binding to eDHFR. Namely, only K32 in eDHFR is located within the “catalyst effective region (CER)” (Figure 1B). We estimate that the CER is a spheric region with a ∼12 Å radius for **1**.^14^ However, the affinity-guided catalysis approach was complicated when we targeted chromatin histones as POI due to the lack of cell-permeable and in-cell stable small-molecule ligands to histones.^13^

Merging the concepts of peptide ligands (Figure 1A) and affinity-guided catalysis (Figure 1B), we herein report designer fusion proteins composed of a peptide ligand and eDHFR, called peptide ligand-inserted eDHFR (PLIED: Figure 1C). POI is selectively acylated by small-molecule catalyst **1** when POI’s specific lysine residues are located within the CER of a POI/PLIED/catalyst ternary complex (Figure 1C). PLIED acts as a universal adapter protein connecting SFSM with POI.

## Results

### PLIEDs for MDM2

To bring POI’s lysine residues and the CER closer together, we sought to insert a peptide ligand of POI into an eDHFR (K32R) mutant. In the eDHFR (K32R) mutant, the sole lysine in eDHFR potentially acylated by **1** was mutated to arginine (Figure 1C) to avoid unnecessary acylation. The PLIED fusion protein may work as a universal adaptor for targeting POI with **1** and enable to promote synthetic lysine acylation at a desired position of POI (Figure 1C). We call this the PLIED/CAT system.

**Figure 2:**
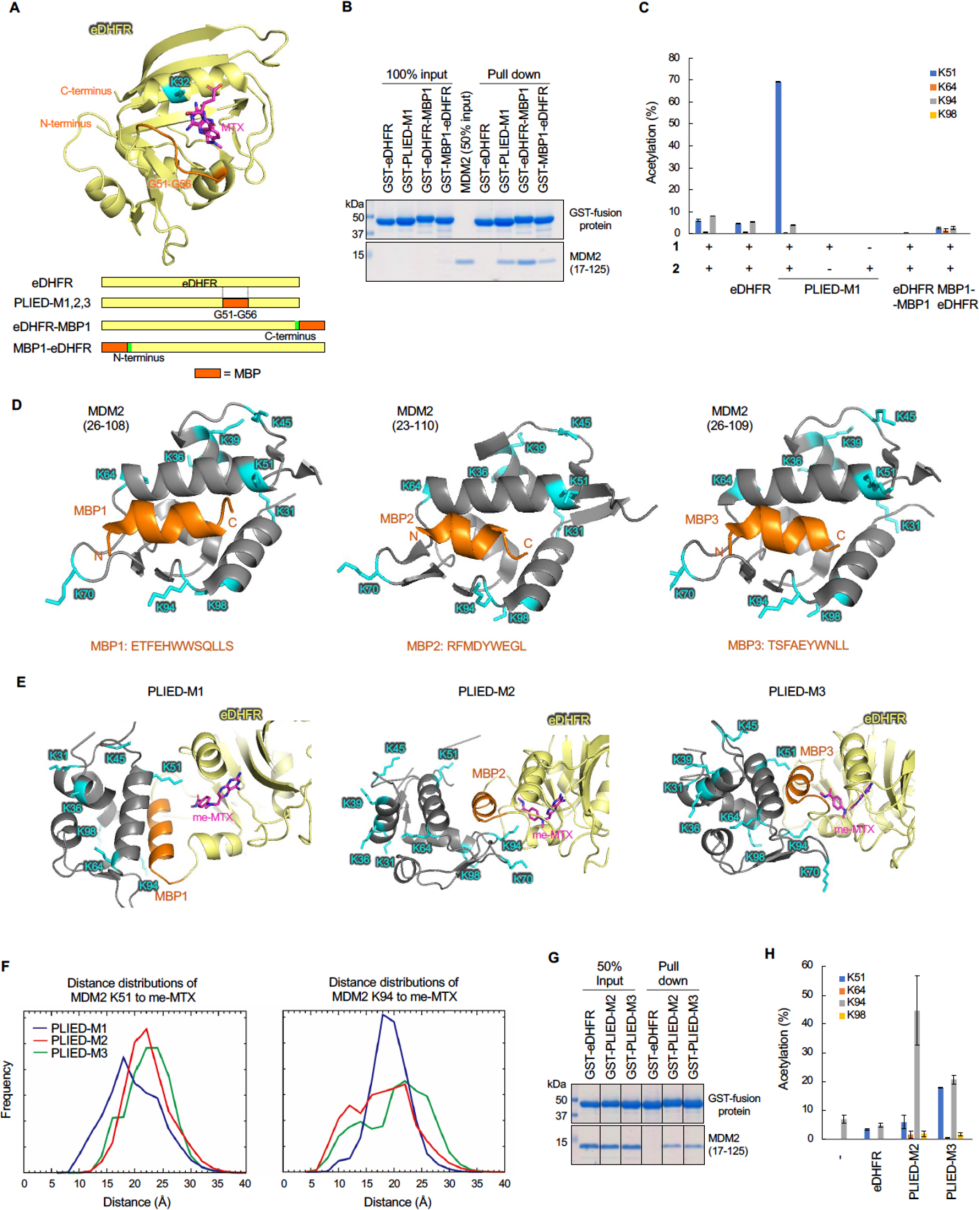
Development of the PLIED/CAT system in vitro. A, Crystal structure of the eDHFR-MTX ligand complex (upper, PDB ID: 1rg7) and schematic representation of MDP-conjugated eDHFR (lower). eDHFR (pale yellow), K32 (cyan), G51–G56 loop region (orange), and MTX (magenta, chemical structure is shown in Figure S1B) are shown and labeled. B, GST-pull down assay with GST-eDHFR derivatives and MDM2. Recombinant human MDM2 (17–125) protein was incubated with glutathione Sepharose-immobilized GST-eDHFR derivatives. After extensive washing, bound proteins were analyzed by SDS-PAGE followed by CBB staining. C, Quantification of MDM2 acetylation by the eDHFR derivatives and TMP-BAHA **1**. Recombinant MDM2 (17–125, 1.4 μM) was incubated with eDHFR, PLIED-M1, eDHFR-MBP1, or MBP1-eDHFR (4 μM) in the presence of TMP-BAHA **1** (10 μM) and acetyl donor **2** (100 μM) at 37 °C for 5 h. Acetylation yields of indicated lysine residues were analyzed by LC-MS/MS. Error bars represent the range of two independent experiments. D, Crystal structure of MDM2 binding with MBP1 (left, PDB ID: 3jzs), MBP2 (middle, PDB ID: 1t4f), or MBP3 (right, PDB ID: 3eqs). MDM2, lysine residues, and MBP are shown in gray, cyan, and orange with label, respectively. E, Modeled structures of PLIED/MDM2 complex. PLIED-M1/MDM2 (left), PLIED-M2/MDM2 (middle), or PLIED-M3/MDM2 (right) complex with eDHFR (pale yellow), me-MTX (magenta, chemical structure is shown in Figure S1B), MDM2 (gray), lysine residues of MDM2 (cyan), and MBP (orange) are shown. F, Distributions of the distances between lysines (K51 or K94) and me-MTX. The distances were measured between the ε-nitrogen atom of the lysines and the center of mass of the benzene ring of me-MTX. The distributions were computed for each of the three cases using the last 9 μs × 15 replicas = 135 μs long trajectories. G, GST-pull down assay with GST-PLIED and MDM2 as in B. H, Quantification of MDM2 acetylation by PLIED/CAT system as in C.

For proof of this concept, we designed PLIED with a previously reported MDM2-binding peptide (MBP1: ETFEHWWSQLLS).^15^ MDM2 is the primary negative regulatory factor of p53 protein. MDM2 binds to p53 and ligates ubiquitin via its E3 ubiquitin ligase moiety. Ubiquitinated p53 is transferred to cytoplasm and degraded by proteasomes.^16^ Therefore, MDM2 maintains stability of the p53 signaling pathway. Several MBPs inhibiting MDM2/p53 binding have been developed so far. We first prepared recombinant MBP1-fused eDHFR where the MBP1 peptide was implanted at the N- or C-terminus of eDHFR, creating MBP1-eDHFR and eDHFR-MBP1, respectively (Figure 2A, Figure S1A). Although both MBP1-eDHFR and eDHFR-MBP1 bound to MDM2 (Figure 2B), any lysine acetylation was not promoted in the presence of **1** and **2**, suggesting that the CER in MBP1-eDHFR and eDHFR-MBP1 did not overlap with lysine residues in MDM2 (Figure 2C). We next assessed a fusion protein with the glycine 51-glycine 56 (G51–G56) loop region of eDHFR replaced by MBP1 with glycine linkers, since the length of MBP1 (∼16.1 Å) is similar to that of the G51–G56 loop (∼15.4 Å), which is located near the ligand-binding pocket of eDHFR (Figure 2A). We referred to this construct as PLIED-M1 and prepared recombinant PLIED-M1 protein from *E. coli* (Figure S1A). PLIED-M1 bound to MDM2 (Figure 2B). Intriguingly, we found that the addition of **1** and **2** to a mixture of MDM2 and PLIED-M1 efficiently promoted lysine 51 (K51) acetylation of MDM2 in 70% yield, but not at other lysine residues (K64, K94, K98, less than 5% yield) (Figure 2A, 2C, Figure S6A), suggesting that only MDM2-K51 was located within the CER when PLIED-M1 bound to MDM2. These data supported our idea that the PLIED/**1** system can promote regioselective lysine acetylation on the target protein.

Next, we hypothesized that lysine residue selectivity of the PLIED/**1** system can be modulated by changing the peptide ligand in PLIED, which may change the position of the CER relative to each lysine residue. We chose two other peptide ligands for MDM2, RFMDYWEGL (MBP2)^17^ and TSFAEYWNLL (MBP3).^18^ Like PLIED-M1, G51–G56 of eDHFR was replaced with MBP2 or MBP3 to create PLIED-M2 or PLIED-M3, respectively. To predict lysine residue selectivity of the PLIED/**1** system, we conducted structural analysis based on reported X-ray structures of methotrexate (MTX)-bound eDHFR (PDB ID: 1rg7) and peptide ligand-bound MDM2 (PDB ID for MBP1: 3jzs, PDB ID for MBP2: 1t4f, PDB ID for MBP3: 3eqs) (Figure 2D, Figure S1B). MTX and TMP bind to the same ligand-binding pocket in eDHFR. Using the modeling software Modeler,^19^ we constructed structural models for each PLIED/**1** system. Then complexes of MDM2 and PLIED proteins were modeled by superimposing the MBP moiety of the PLIED proteins with MBP bound to MDM2 (Figure 2E). We then examined distributions of the distance between the ligand-binding pocket of PLIED and MDM2 lysine residues by molecular dynamics (MD) simulation of the PLIED-MDM2 complex model. The MD simulation suggested that, compared to PLIED-M1, the ligand-binding pocket of PLIED-M2 was farther away from K51 and closer to K94 (Figure 2F). The distribution of the ligand-binding pocket of PLIED-M3 was intermediate between that of PLIED-M1 and PLIED-M2 (Figure 2F). These data suggested that the PLIED-M2/CAT system may acetylate MDM2-K94 rather than MDM2-K51. We then prepared recombinant PLIED-M2 and PLIED-M3 proteins from *E. coli* (Figure S1C). Remarkably, PLIED-M2/**1** efficiently and selectively promoted MDM2-K94 acetylation in the presence of **2**, whereas both MDM2-K51 and MDM2-K94 acetylation were promoted by PLIED-M3/**1** (Figure 2G, 2H, Figure S6B). Taken together, our data indicate that lysine residue-selectivity of the PLIED/**1** system is predictable in an atomic level from MD simulations of the POI/PLIED/**1** ternary complex.

### PLIEDs for Histones

**Figure 3:**
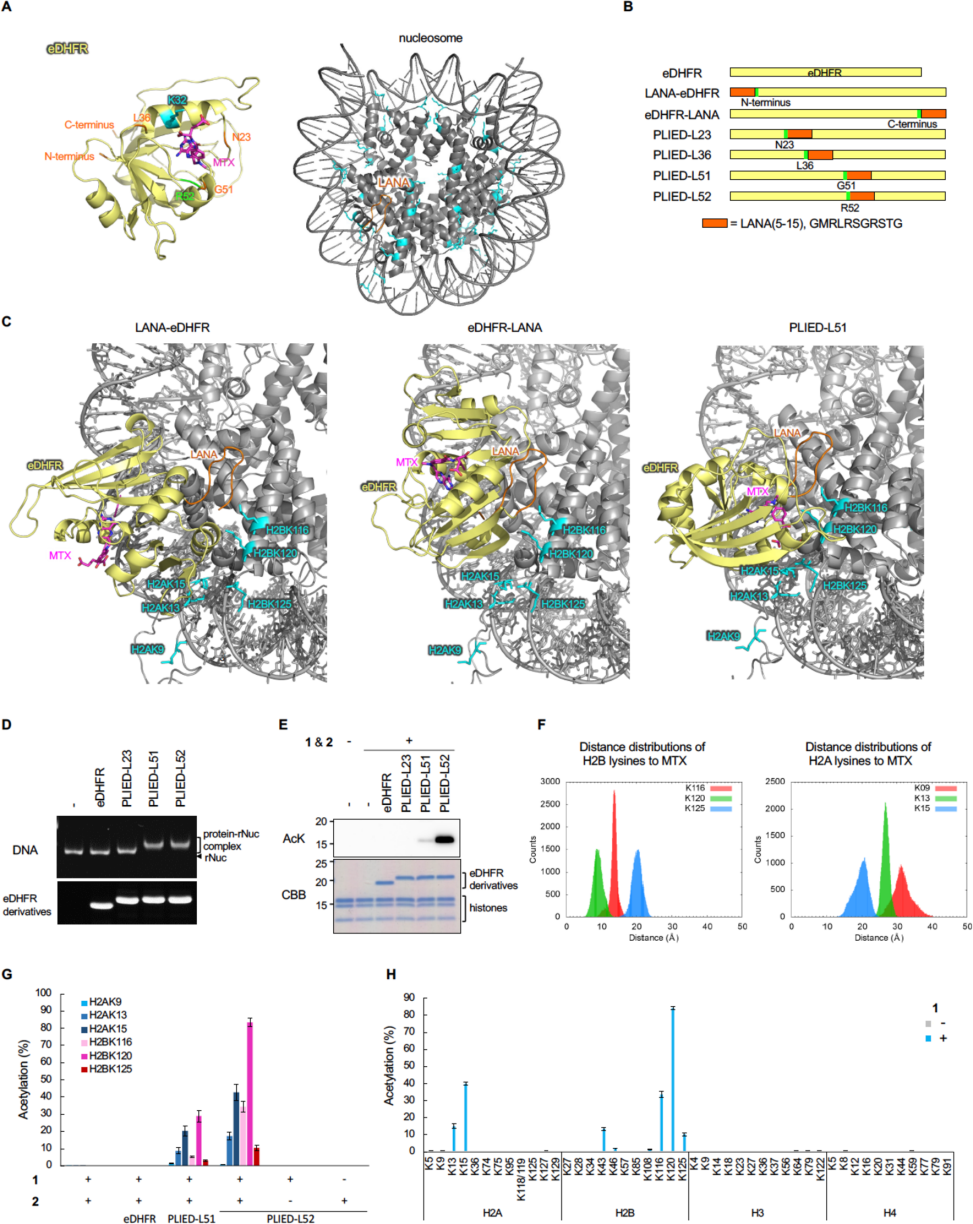
Development of a histone acylation system. A, Crystal structure of the eDHFR-MTX ligand complex (left, PDB ID: 1rg7). eDHFR (pale yellow), MTX (magenta), and amino acid residues of interest (green, orange, and cyan) are shown and labeled. Crystal structure of the nucleosome-LANA (5–15) complex (right, PDB ID: 5gtc). LANA and lysine residues of histones are shown in orange with label and in cyan, respectively. B, Schematic representation of LANA-conjugated eDHFR. C, Modeled structures of LANA-inserted eDHFR-nucleosome complex. The LANA (5–15) was implanted at the N-terminus (left), the C-terminus (middle), or between G51 and R52 (right) of eDHFR. eDHFR, LANA, MTX, and lysines are shown in pale yellow, orange, magenta, and cyan with labels, respectively. D, Electrophoretic mobility shift assay of PLIED-bound nucleosomes. Recombinant nucleosomes (0.2 μM) were incubated with the indicated proteins (10 μM). The samples were analyzed by 6% nondenaturing PAGE in 0.5× TBE buffer, and DNA was visualized by ethidium bromide staining. The positions of recombinant nucleosomes (rNuc) are shown. For confirmation of protein levels of eDHFR derivatives, the samples were analyzed by SDS-PAGE, and proteins were visualized by Oriole staining. Representative data of two independent experiments are shown. E, Histone acetylation with PLIED/**1** systems. Recombinant nucleosomes (0.35 μM) were incubated with eDHFR or PLIED protein (2 μM), acetyl donor **2** (100 μM), and TMP-BAHA **1** (5 μM) at 37 °C for 5 h. The lysine acetylation was detected by immunoblotting using anti-acetyl lysine (AcK) antibody. Proteins were visualized by CBB staining. Representative data of two independent experiments are shown. F, Distributions of the distances between lysines (H2A K9, K13 and K15, and H2B K116, K120 and K125) and MTX. Measured distances were between the ε-nitrogen atom of the lysines and the center of mass of the benzene ring of MTX (chemical structure is shown in Figure S1B). The distributions were computed using the last 50 ns of the MD trajectories. G, Quantification of histone acetylation by PLIED/**1** systems. Recombinant nucleosomes (0.35 μM) were incubated with eDHFR or PLIED protein (2 μM), acetyl donor **2** (100 μM), and TMP-BAHA **1** (5 μM) at 37 °C for 5 h. Acetylation yields of the indicated residues, which were predicted to be close to the TMP-binding pocket of PLIED-L51, were analyzed by LC–MS/MS. H, Lysine residue selectivity of histone acetylation by PLIED-L52. Recombinant nucleosomes (0.35 μM) were incubated with PLIED-L52 (2 μM) and acetyl donor **2** (100 μM) with or without TMP-BAHA **1** (5 μM) at 37 °C for 5 h. Acetylation yield of indicated lysine residues were analyzed by LC–MS/MS. For LC–MS/MS data, error bars represent the range of two independent experiments (G, H).

We next focused on construction of the PLIED/**1** system for physiologically relevant acetylated proteins, histones. So far, there are no small molecule ligands for histones. As a peptide ligand for histones, we used the N-terminal fragment of LANA^20^ (residues 5–15, corresponding to the minimum histone-binding region of LANA, hereafter referred to as LANA). As described above, we conducted structural analysis based on reported X-ray structures of MTX-bound eDHFR (PDB ID: 1rg7) and LANA-bound nucleosome (PDB ID: 5gtc) (Figure 3A). Initially, we constructed structural models for LANA-fused eDHFR where the LANA peptide was implanted at the N- or C-terminus of eDHFR, creating LANA-eDHFR and eDHFR-LANA, respectively (Figure 3B). However, no models were found where the MTX ligand bound to eDHFR is directing to lysine residues in histones without collision between eDHFR and nucleosome. This result suggests that the CER in LANA-eDHFR and eDHFR-LANA cannot overlap with histone lysine residues (Figure 3C, left and middle panels, and Figure S2A). We next assessed a fusion protein with LANA inserted between glycine 51 (G51) and arginine 52 (R52) of eDHFR, since LANA formed hairpin-like structure (Figure 3A) and the distance between the N- and C-termini of LANA was short (∼5.6 Å). We refer to this construct as PLIED-L51 (PLIED with LANA at G51). Intriguingly, we did find modeled structures where the MTX ligand bound to PLIED-L51 was oriented toward lysine residues in H2A and H2B (Figure 3C, right panel, and Figure S2A), suggesting that the CER in PLIED-L51 may overlap with some lysine residues in histones.

On the basis of the modeling analysis, we investigated whether the PLIED-L51/**1** system could promote histone acetylation. We expressed and purified recombinant eDHFR, eDHFR-LANA, LANA-eDHFR, and PLIED-L51 proteins in *E. coli.* In addition to PLIED-L51, we constructed PLIED-L23, -L36, and -L52, in which the insertion site of LANA is located in the loop region near the ligand-binding pocket of eDHFR (Figure 3A, 3B). We successfully purified these recombinant PLIED proteins except for PLIED-L36, which went to the insoluble fraction during purification step (Figure S2B, S2C). Electrophoretic mobility shift assay showed that PLIED-L51 and PLIED-L52, but not eDHFR, bound to recombinant nucleosome, indicating that the LANA peptide in these constructs was functional (Figure 3D). In contrast, LANA-eDHFR, eDHFR-LANA, and PLIED-L23 did not bind to recombinant nucleosome, suggesting that, for reasons unknown, the LANA peptide in these constructs may not be exposed to the solvent (Figure 3D and Figure S2D). Therefore, the insertion of LANA to an appropriate position of eDHFR is important to construct the functional PLIED/CAT system bearing properly folded eDHFR and a peptide ligand. Then we examined whether the PLIED/**1** system could acetylate histones in vitro. Recombinant PLIED proteins were mixed with recombinant nucleosomes containing a histone octamer (two copies of H2A, H2B, H3, and H4) and DNA, in the presence or absence of **1** and **2**, and histone acetylation was evaluated by immunoblot analysis using anti-pan acetyl-lysine (AcK) antibody. The addition of **1** and **2** clearly promoted histone acetylation in the presence of PLIED-L52 (Figure 3E). Slight histone acetylation was detected for PLIED-L51, while no detectable histone acetylation was observed for PLIED-L23 or eDHFR (Figure 3E). These results indicate that the CER overlapped with lysine residues in histones for PLIED-L52 or PLIED-L51.

We next addressed which lysine residues in histones were acetylated by the PLIED/**1** system. First, we examined distributions of the distance between the ligand-binding pocket of PLIED and histone lysines by MD simulation of the PLIED-L51-nucleosome complex model. The MD simulation suggested that histone H2B lysine 120 (H2BK120) is the most proximal lysine to the ligand-binding pocket of PLIED-L51 (distance between the ε-nitrogen atom of lysine and the center of mass of the benzene ring of MTX was 5 to 14 Å with a peak at ∼8.8 Å, Figure 3F). The second proximal lysine was histone H2B lysine 116 (H2BK116) (8 to 17 Å with a peak at ∼14.9 Å), and the third was histone H2B lysine 125 (H2BK125) or histone H2A lysine 15 (H2AK15) (13 to 24 Å with a peak at ∼20 Å). Then we conducted acetylation reactions of recombinant nucleosome by the PLIED/**1** system in test tubes and quantified the acetylation stoichiometry of those lysine residues. Consistent with the MD simulation results, the PLIED-L52/CAT system acetylated H2BK120 most efficiently (>80% yield) (Figure 3G, 3H, Figure S6C). H2BK116 and H2AK15 were moderately acetylated (30–40% yield), and H2BK125 and H2AK13 were slightly acetylated (<20%) (Figure 3G, 3H). In contrast, none of lysine residues in H3 or H4 was significantly acetylated by the PLIED-L52/**1** system (Figure 3H). When PLIED-L51 was used, a similar synthetic histone acetylation pattern was observed but with lower yields than PLIED-L52 (Figure 3G). These results indicated that, when PLIED-L52 interacted with nucleosomes, PLIED-L52-bound catalyst was most proximal to H2BK120 and thus can promote efficient and regioselective synthetic histone acetylation. Furthermore, these data demonstrated again that the lysine residue-selectivity of the PLIED/CAT system is predictable in an atomic level from MD simulations of the POI/PLIED/CAT ternary complex.

### In-Cell Histone Acylation by PLIED/**1** System

**Figure 4:**
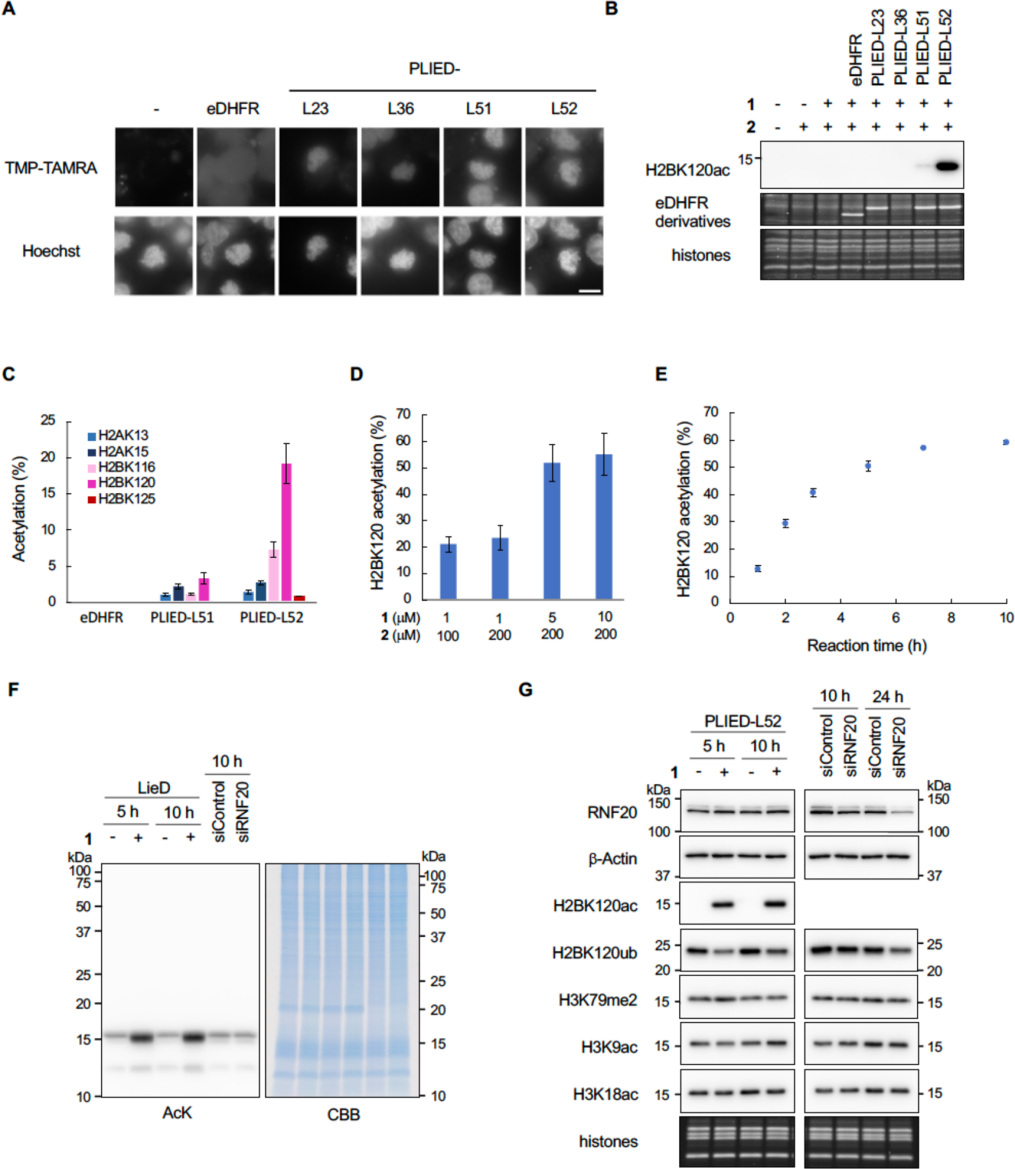
In-cell histone acetylation reaction by PLIED/1. A, Subcellular localization of PLIED. eDHFR-FLAG- or PLIED-FLAG-transfected HEK293T cells were treated with nocodazole (330 nM) for 4 h, followed by TMP-TAMRA (10 μM, chemical structure is shown in Figure S3A) with nocodazole for 1 h. DNA was stained with Hoechst 33342 to visualize chromatin distribution. Representative images of mitotic cells are shown. Scale bar, 10 μm. B, In-cell histone acetylation by PLIED/**1** systems. eDHFR-FLAG- or PLIED-FLAG-transfected HEK293T cells were incubated with or without acetyl donor **2** (100 μM) and TMP-BAHA **1** (1 μM) at 37 °C for 5 h. Whole-cell extracts were immunoblotted with anti-H2BK120ac antibody. The expressed eDHFR derivatives and histones were visualized by Oriole staining. Representative data of two independent experiments are shown. C, Acetylation yields of indicated lysine residues on histones from PLIED/**1**-treated HEK293T cells as in B. The yield was determined by LC–MS/MS. D, Optimization of histone acetylation by PLIED-L52. PLIED-L52-FLAG-transfected HEK293T cells were incubated with TMP-BAHA **1** and acetyl donor **2** at the indicated concentrations at 37 °C for 5 h. E, Time course analysis of histone acetylation by PLIED-L52/**1**. PLIED-L52-FLAG-transfected HEK293T cells were incubated with TMP-BAHA **1** (5 μM) and acetyl donor **2** (200 μM) at 37 °C for the indicated time. Acetylation yields were analyzed by LC–MS/MS. For LC–MS/MS data, error bars represent the range of two independent experiments (C–E). F, Histone-selectivity of PLIED-L52/**1**-mediated acetylation. PLIED-L52-FLAG-transfected HEK293T cells were incubated with or without TMP-BAHA **1** (5 μM) in the presence of acetyl donor **2** (200 μM) at 37 °C for 5 or 10 h. For knockdown of RNF20, HEK293T cells were transfected with control or RNF20 siRNA and harvested after 10 h. The lysine acetylation levels of proteins in the whole-cell extract were detected by immunoblotting using anti-AcK antibody. Proteins were visualized by CBB staining. Representative data of two independent experiments are shown. G, Immunoblotting analysis of the PLIED-L52/**1**-mediated in-cell acetylation. HEK293T cells were treated as in F. To assess levels of RNF20, whole-cell extracts were immunoblotted with anti-RNF20. β-Actin was used as a loading control. To assess levels of histone PTMs, histones were acid-extracted from the cells and analyzed by immunoblotting with the indicated antibodies. The loading amounts of histones were visualized with Oriole staining. Representative data of two independent experiments are shown.

Next, we studied the PLIED/**1** system in living cells. First, we confirmed that PLIED-L51 and PLIED-L52, expressed in HEK293T cells, bound to chromatin by visualizing these proteins with TMP-conjugated TAMRA (Figure 4A and Figure S3A). PLIED-L23, PLIED-L36, LANA-eDHFR, or eDHFR-LANA weakly bound to chromatin, while eDHFR did not (Figure 4A and Figure S3B). Second, we added **1** and **2** to the culture media of HEK293T cells and examined whether histone acetylation was promoted. As expected, H2BK120 acetylation was significantly promoted by **1** and **2** in the presence of PLIED-L52 (Figure 4B, 4C and Figures S4 and S6D). H2BK120 acetylation was slightly promoted in the presence of PLIED-L51, but not in the presence of PLIED-L23, PLIED-L36, LANA-eDHFR, eDHFR-LANA, or eDHFR (Figure 4B, 4C and Figure S3C). After optimizing concentrations of **1** and **2**, the yield of H2BK120 acetylation increased up to ca. 60% using 5–10 μM **1** and 200 μM **2** (Figure 4D). Time-course analysis of the reaction showed that the yield of H2BK120 acetylation increased linearly until ∼3 h and reached a plateau at 5–7 h (Figure 4E). The overexpression of eDHFR within this time scale did not affect cell viability in HEK293T and HeLa S3 cells (Figure S3D). To evaluate whether the PLIED-L52/**1** system selectively promoted histone acetylation in living cells, we conducted immunoblot analysis using anti-pan acetyl-lysine antibody. As expected, the addition of **1** and **2** selectively promoted histone acetylation, and acetylation of other cellular proteins was not detected (Figure 4F). Analyses using site-specific anti-acetyl lysine antibodies confirmed that the PLIED-L52/**1** system promoted H2BK120 acetylation, but not H3-tail acetylation (Figure 4G).

Synthetic H2BK120 acetylation in living cells mediated by the chemical catalyst system can work as a protecting group at H2BK120 and inhibit H2BK120 ubiquitination (H2BK120ub) catalyzed by histone-ubiquitination enzymes.^13^ Accordingly, immunoblot analysis showed that after 5 h, the global level of H2BK120ub was significantly reduced by PLIED-L52/**1**-dependent H2BK120 acetylation (Figure 4G). This approach reduced H2BK120ub much more rapidly than RNAi-mediated knockdown of the H2B E3 ubiquitin ligase, ring finger protein 20 (RNF20)^21^ (Figure 4G). These data indicate that the PLIED/**1** system efficiently worked in living cells and that synthetic histone acetylation by the system inhibited H2B ubiquitination.

**Figure 5:**
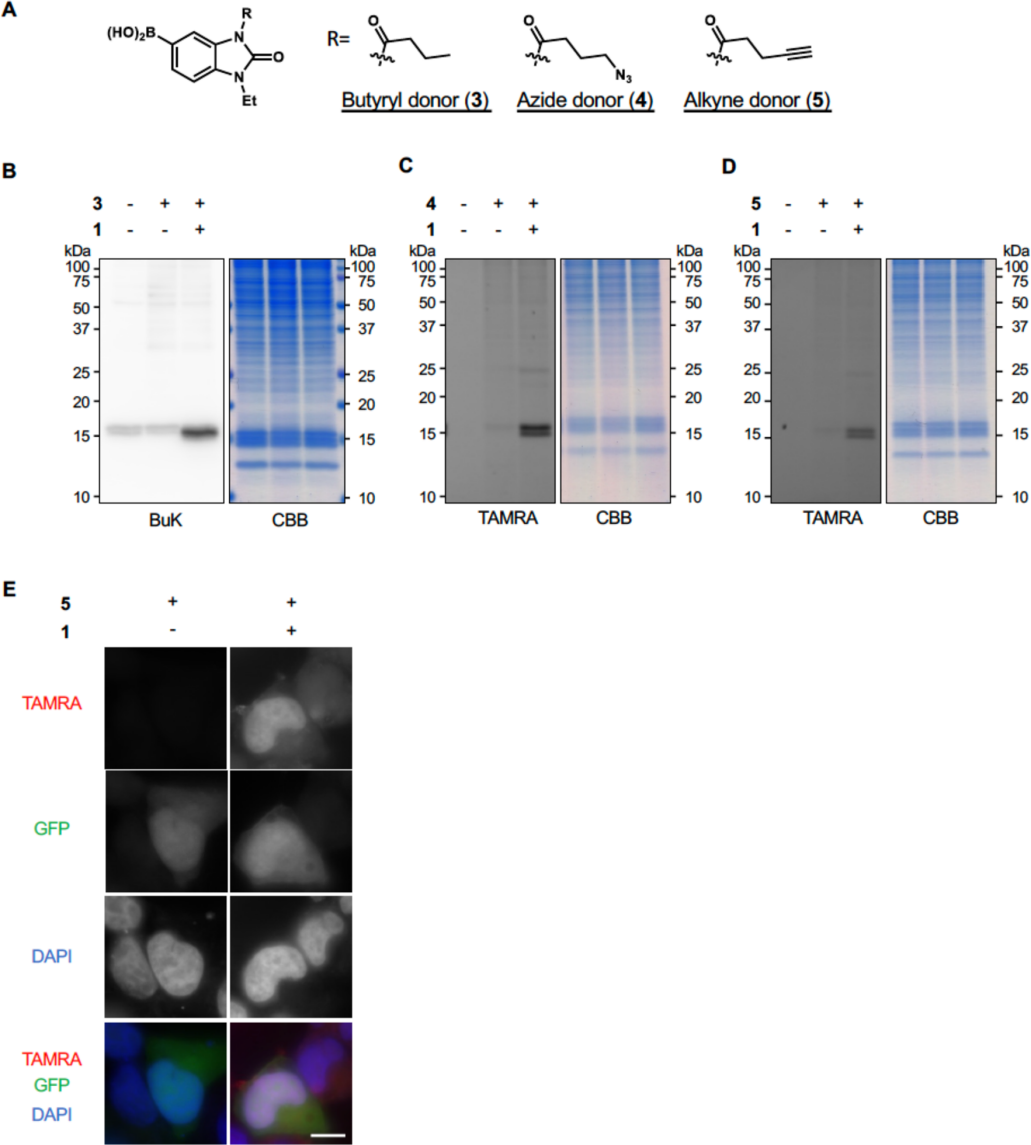
Histone acylation by PLIED-L52/1. A, Chemical structures of butyryl donor **3**, azide donor **4**, and alkyne donor **5**. B–D, Histone-selectivity of PLIED-L52/**1**-mediated lysine acylations. PLIED-L52-FLAG-transfected HEK293T cells were incubated with or without TMP-BAHA **1** (5 μM) and acyl donors (200 μM) at 37 °C for 5 h. To detect butyrylation, whole-cell extracts were analyzed by immunoblotting with anti-butyryl lysine (BuK) antibody. To detect acylation containing azide or alkyne, acylated lysines were labeled with TAMRA-alkyne or TAMRA-azide, respectively, by Cu(I)-catalyzed azide–alkyne cycloaddition reaction. The fluorescence detection is shown (TAMRA). Proteins were visualized by CBB staining. E, PLIED-L52-FLAG- and EGFP-cotransfected HeLaS3 cells were incubated with or without TMP-BAHA **1** (5 μM) and alkyne donor **5** (100 μM) at 37 °C for 5 h. The cells were fixed, permeabilized, and blocked with 3% BSA, and then acylated lysines were labeled with TAMRA-azide by Cu(I)-catalyzed azide–alkyne cycloaddition reaction. The nuclei were visualized by DAPI staining.

Catalyst **1** can promote not only acetylation but also other acylations, including non-natural ones, simply by changing acyl donors.^14^ We prepared acyl donors with butyryl **3**, azide **4**, alkyne **5**, and alkyne with diazirine **6** groups (Figure 5A, Figure S5A). First, we confirmed that MDM2 and histones were acylated by the PLIED/**1** system using acyl donors **3**–**6** in test tubes (Figure S5B, S5C). We then conducted in-cell acylation reactions with PLIED-L52/**1**. As expected, the addition of **1** and the acyl donors to HEK293T cells expressing PLIED-L52 selectively promoted histone acylation in all cases (Figure 5B–D). We envisioned that this system could be used as a unique tool to visualize endogenous histones through click chemistry of the azide- or alkyne-modified histones with fluorescent dyes, which would be useful for chromosome analysis and genetic diagnostics.^22^ HeLa S3 cells, expressing PLIED-L52 and GFP, were treated with **1** and alkyne-containing acyl donor **5**, and acylated histones were labeled with azide-conjugated TAMRA. While GFP signals were distributed throughout whole cells, TAMRA signals were colocalized with chromosomal DNA (Figure 5E), suggesting that histones were exclusively labeled by the PLIED-L52/**1** system.

## Discussion

In this study, we developed engineered PLIED proteins, in which peptide ligands of POI are inserted into eDHFR at suitable positions, as universal adaptors for targeting POI with cell-permeable and in-cell stable SFSM (e.g., chemical catalyst **1** in this study). In the PLIED/**1** system, PLIED binds to POI through the peptide moiety, properly orienting its eDHFR moiety, which then recruits TMP-conjugated chemical catalyst **1** to POI. We demonstrated that POI (MDM2 and histone) are post-translationally and synthetically acylated at specific lysine residues. In theory, any peptide ligands can be inserted into eDHFR to create new PLIED proteins for directing SFSM to POI. For efficient installation of PTMs, POI’s lysine residues should be located within the CER, a spheric region with a ∼12 Å radius for the chemical catalyst, when PLIED bound to POI. As demonstrated in this study, MD simulations of the POI/PLIED/SFSM ternary complex help designing new PLIED proteins and predicting its target residue-selectivity in an atomic level. It is noteworthy that, by changing the peptide ligand for MDM2, lysine residue-selectivity of the PLIED/**1** system was converted from K51-selective to K94-selective.

The PLIED/**1** system allowed us to install synthetic H2BK120 acylation using cell-permeable and stable (i.e., peptidase-resistant) small molecule catalyst **1** in living cells. We showed that in-cell synthetic histone acetylation by the PLIED/**1** system cross-talked with other histone marks, such as H2BK120 ubiquitination, which are intimately linked with active gene transcription.^23^ In this sense, the PLIED/**1** system functions as an artificial histone acetyltransferase and intervenes in the epigenome network in living cells. While histone acetyltransferases utilize acyl-CoAs for histone acylation, the PLIED/**1** system uses cell-permeable synthetic acyl donors **2**–**5** containing a boronic acid. In principle, the PLIED/**1** system can introduce any acyl types, natural or unnatural, and thus the acylation scope of the PLIED/**1** system is wider than that of histone acetyltransferase enzymes. Using acyl donors containing biorthogonal moieties, such as azides or alkynes, the PLIED/**1** system can be used to attach probes to endogenous histones via click chemistry. To the best of our knowledge, this is the first example of probing endogenous histones in living cells. As demonstrated in this study, conjugation of a fluorescent dye can visualize endogenous histones, thus constituting a useful method for chromosome imaging and genetic diagnostics. Conjugation with other probes, such as a photo-cross-linker,^24^ will also provide an attractive new tool in chromosome biology and epigenome study.

Synthetic acetylation at H2BK120 in living cells mediated by the PLIED-L52/**1** system can work as a protecting group and inhibit ubiquitination at H2BK120. As a method for H2BK120ub inhibition, the PLIED-L52/**1** system has at least two advantages over RNAi-mediated knockdown of H2BK120ub enzymes, such as RNF20. First, because nonhistone proteins, such as Eg5,^25^ are also targeted by RNF20, RNF20 inhibition leads to phenotypes not related to H2BK120ub. In contrast, the PLIED-L52/**1** system is histone-selective and thus its phenotypes are likely linked with H2BK120ub inhibition. It should be noted, however, that other lysine residues proximate to H2BK120, such as H2BK116, were also acetylated by the PLIED-L52/**1** system, which may contribute H2BK120ub inhibition. Second, while RNAi-mediated knockdown needs more than 1 day for H2BK120ub reduction, the PLIED-L52/**1** system can inhibit H2BK120ub within several hours, which may help analyzing the functions of H2BK120ub in dynamic cellular processes, such as cell cycle progression. So far, various enzyme mimics (also called “artificial enzymes”), such as nanozymes, have been extensively developed to address the limitations of natural enzymes.^26^ The merging of chemical catalysis with MD-supported protein engineering developed in this study demonstrates a novel concept: artificial enzymes enabling diverse reactivity, with physiologically relevant selectivity, in living cells.

## Methods

### Modeling Docking Pose and Molecular Dynamics Simulation for the PLIED-MDM2 Complex Model

Methotrexate (MTX)-bound eDHFR and MDM2 structures were taken respectively from the Protein Data Bank: PDB ID 3dau (eDHFR + MTX) and 3jzs (MBP1), 1t4f (MBP2), and 3eqs (MBP3). First, the carboxyl group in MTX was methylated to neutralize the charge of MTX because it may excessively interact with lysines of MDM2. Each of GGG-peptide-GGG bound to MDM2 was replaced with a loop of G51 to G56 of the eDHFR using Modeler.^19^ For each of the three peptide-fused eDHFR (referred to PLIED-M1–3 hereafter), 100 structures were generated where four residues on both sides of the implanted peptide were allowed to move but the other part of the MDM2-eDHFR-me-MTX complex was fixed. The structure with the minimum energy was selected as the initial structure for the following molecular dynamics simulation.

To examine the accessibility of the me-MTX and K51 or K94, we carried out canonical molecular dynamics (MD) simulations using pmemd module of amber16^27^ with the AMBER ff99SB,^28^ ff99ions08,^29^ and generalized AMBER force field (GAFF)^30^ force field. The force field parameters for me-MTX were prepared using the Antechamber package in AmberTools. The charges of me-MTX were derived by the quantum mechanical optimization with HF/6-31G* level followed by the Restrained Electrostatic Potential (RESP) with HF/6-31G* calculation. The system of the MDM2-eDHFR-me-MTX complex was placed in a rectangular box ∼ 108 Å × 108 Å × 108 Å at 0.15 M NaCl. In this box, all the atoms of the complex were separated more than 20 Å from the edge of the box. Then ∼38 000 TIP3P water molecules^31^ were added to surround the systems. In total, the system comprised ∼120 000 atoms. For each the three cases, 16 10 μs long MDs were independently conducted with the same initial structure but with different initial velocity assignment (10 μs × 16 replicas = 160 μs) at a constant pressure of one bar and a temperature of 300 K. The dielectric constant used was 1.0, and the van der Waals interactions were evaluated with a cutoff radius of 9 Å. The particle mesh Ewald (PME) method^32^ was used for the electrostatic interactions, in which the charge grid size was chosen to be close to 1 Å. The charge grid was interpolated using a cubic B-spline of the order of four with the direct sum tolerance of 10^–5^ at the 9 Å direct space cutoff. Langevin dynamics algorithm with collision frequency of 2 ps^–1^ was utilized to control the temperature and pressure of the system. The coupling times for the temperature and pressure control were both set at 1 ps^–1^. The SHAKE algorithm^33^ was used to constrain all the bond lengths involving hydrogen atoms. The leapfrog algorithm with a time step of 2 fs was used throughout the simulation to integrate the equations of motion. The system was first heated from 0 to 300 K within 10 ns during which the molecules were fixed with decreasing restraints and the water molecules and ions were allowed to move. The conformational snapshot was saved every 100 ps. For each of the three cases, 1 replica out of 16 replicas showed a significant dislocation of me-MTX from the original binding site on eDHFR; the trajectory of the replica was thus excluded from the further analyses. Using snapshots of the last 9 μs × 15 replicas = 135 μs trajectories, we examined distributions of the distance between the nitrogen atom of lysines of interest and the center of mass of the benzene ring of me-MTX.

### Modeling Docking Pose and Molecular Dynamics Simulation for the PLIED-LANA Complex Model

MTX-bound eDHFR and LANA-bound nucleosome structures were taken from the Protein Data Bank whose ID were 1rg7 and 5gtc. The LANA peptide was implanted at the N- and C-termini and G51 of eDHFR using Modeler.^19^ For each of the three cases, 100 structures were generated and the models which did not have any collision with the nucleosome were selected. No model was found where the MTX ligand bound to eDHFR is directing to the H2A K9, K13, K15 or H2B K116, K120, K125 when LANA was implanted at the N- and C-termini. In the models in which the LANA was inserted at G51, we found structures in which MTX was accessible to H2A and H2B lysines. Selecting two structures manually, we further searched for a stable docking pose using Simulated Annealing (SA) molecular dynamics of amber 16^27^ with the AMBER ff99SB^28^ and ff99bsc0^34^ force field. SA of the nucleosome-eDHFR complex was performed in vacuum using the distance-dependent dielectric constant of 4.0*r* with the value of *r* in angstroms. Residues outside the LANA peptide, S49–G52 and G62–P64, were set to move freely. Strong restraints were applied to the other parts to prevent the structure from collapsing at a high temperature: (1) Harmonic restraints with a force constant of 10 kcal/mol/Å^2^ were applied to the heavy atoms in the nucleosome. (2) Distance restraints with a force constant of 1000 kcal/mol/Å^2^ were applied to the heavy atoms in eDHFR, whose distances are less than 5 Å, at their initial values. (3) Harmonic restraints with a force constant of 10 kcal/mol/Å^2^ were applied to the LANA peptide in eDHFR for maintenance of the interaction between the LANA peptide in eDHFR and nucleosome. Nonbonded interactions were evaluated with a cutoff radius of 12 Å, and a time-step of 0.5 fs was used. The system was heated from 0 to a high temperature of about 1000 K during the first 10 ps and was then equilibrated for 10 ps. The equilibrated system was then gradually cooled for 80 ps from the high temperature to 0 K. The SA was repeated 100 times, and the resulting coordinate sets were stored as possible conformations of the complex at local minimum energy regions. Each of the 100 conformations was minimized for 500 steps using the steepest descent algorithm followed by 9500 steps of the conjugate gradient algorithm. Harmonic restraints with a force constant of 10 kcal/mol/Å^2^ were applied to all the heavy atoms of the nucleosome–eDHRF complex. Then the structure with the minimum energy was selected as the initial structure for the following molecular dynamics simulation.

Lastly, we examined the accessibility of the MTX and lysines by 120-ns-long canonical molecular dynamics simulation based on the minimum energy structure. The MD was carried out using pmemd module of amber16 with the AMBER ff99SB,^28^ ff99bsc0,^34^ ff99ions08,^29^ and generalized AMBER force field (GAFF).^30^ The minimum energy structure was placed in an aqueous medium with MTX. The force field parameters for MTX were prepared using the Antechamber package in AmberTools. The charges of MTX were derived by the quantum mechanical optimization with B3LYP/6-31G* level followed by the Restrained Electrostatic Potential (RESP) with HF/6-31G* calculation. The system of the nucleosome-eDHFR-MTX complex was placed in a rectangular box ∼ 140 Å × 140 Å × 150 Å. In this box, all the atoms of the complex were separated more than 15 Å from the edge of the box. To neutralize the charges of the system, potassium ions were placed at positions with large negative electrostatic potential. Then ∼ 75 000 TIP3P water molecules^31^ were added to surround the systems. In total, the system comprised ∼250 000 atoms.

The MD simulation was carried out at a constant pressure of one bar and a temperature of 300 K. The dielectric constant used was 1.0, and the van der Waals interactions were evaluated with a cutoff radius of 9 Å. The particle mesh Ewald (PME) method^32^ was used for the electrostatic interactions, in which the charge grid size was chosen to be close to 1 Å. The charge grid was interpolated using a cubic B-spline of the order of four with the direct sum tolerance of 10^–5^ at the 9 Å direct space cutoff. Langevin dynamics algorithm with collision frequency of 2 ps^–1^ was utilized to control the temperature and pressure of the system. The coupling times for the temperature and pressure control were both set at 1 ps^–1^. The SHAKE algorithm^33^ was used to constrain all the bond lengths involving hydrogen atoms. The leapfrog algorithm with a time step of 2 fs was used throughout the simulation to integrate the equations of motion. The system was first heated from 0 to 300 K within 1 ns during which the molecules and potassium ions were fixed with decreasing restraints and the water molecules were allowed to move. A conformational snapshot was saved every 1 ps. Using snapshots of the last 50 ns trajectory, we examined distributions of the distance between the nitrogen atom of lysines of interest and the center of mass of the benzene ring of MTX.

### Preparation of Recombinant Proteins

The pGEX-6P-2-eDHFR, -eDHFR derivatives, or -human MDM2 (hMDM2) expression plasmids were used to transform *E. coli* BL21C+ competent cells. Plasmids are listed in Table S1. The cells carrying the expression plasmids were precultured in LB medium containing ampicillin (50 μg/mL) and chloramphenicol (30 μg/mL) overnight at 37 °C. The cell culture was diluted 200- to 500-fold into LB medium containing ampicillin and chloramphenicol, cultured at 37 °C until the OD_600_ reached approximately 0.4, and then isopropyl β-d-thiogalactoside (IPTG; 0.2 mM) was added to induce protein expression. After overnight culture at 16 °C, the cells were harvested by centrifugation. The cell pellet was resuspended in solubilization buffer (40 mM Tris-HCl (pH 7.5), 0.5 mM EDTA, 0.5% Triton X-100, 100 mM NaCl, 1 mM PMSF), and the suspension was sonicated and centrifuged at 10 000 g for 15 min. Expressed GST-tagged protein in the supernatant was mixed with Glutathione Sepharose 4B (GE Healthcare, 17-0756-01), and the mixture was incubated at 4 °C for 1–3 h with rotation. GST-tagged protein-bound beads were washed with solubilization buffer twice and PreScission buffer (50 mM Tris-HCl (pH 7.5), 1 mM EDTA, 100 mM NaCl, 1 mM DTT) once. The protein-bound beads were incubated with PreScission protease (GE Healthcare, 27–0843–01) at 4 °C for at least 20 h to cleave GST from expressed proteins and elute the proteins from beads. The isolated proteins were concentrated by ultrafiltration using Amicon Ultra 10K (Millipore), and the protein concentrations were determined by Bradford assay (TaKaRa). Glutathione Sepharose-immobilized GST-eDHFR derivatives determined the protein concentrations from a standard curve with known concentrations of bovine serum albumin following SDS-PAGE and CBB staining before treatment with PreScission protease. Recombinant nucleosomes were reconstituted and purified as described previously.^11,35^

### GST-Pull Down Assay

Glutathione Sepharose-immobilized GST-eDHFR or -eDHFR derivatives (2.5 μM for Figure 2B, 5 μM for Figure 2G) was incubated with recombinant hMDM2 (5 μM for Figure 2B, 10 μM for Figure 2G) in 50 mM Tris-HCl (pH 7.5), 100 mM NaCl, 0.01% Triton X-100, 0.4–0.45 mM DTT, and 0.4–0.45 mM EDTA at 4 °C for 2–3 h. After washing the beads in buffer (50 mM Tris-HCl (pH 7.5), 100 mM NaCl, 0.01% Triton X-100) three times, bound proteins were analyzed by SDS-PAGE followed by CBB staining.

### Gel Electrophoretic Mobility Shift Assay

Recombinant nucleosomes (20 ng/mL for DNA concentration, approximately 0.2 μM) were incubated with eDHFR or eDHFR derivatives in 26 mM Tris-HCl (pH 7.5) buffer containing 1 mM DTT, 20 mM NaCl, and 0.2 mM EDTA at 30 °C for 1 h. For Figure S2D, nucleosomes and proteins were incubated in 32 mM Tris-HCl (pH 7.5) buffer containing 1 mM DTT, 40 mM NaCl, and 0.4 mM EDTA at 30 °C for 30 min. The samples were analyzed by nondenaturing 6% PAGE in 0.5× Tris-borate EDTA (TBE) buffer (45 mM Tris base, 45 mM boric acid, 1 mM EDTA). DNA was visualized by ethidium bromide staining. To confirm the protein levels, the samples were boiled with SDS sample buffer and analyzed by SDS-PAGE followed by Oriole staining (Bio-Rad).

### In Vitro Acylation Assay

Recombinant hMDM2 (1.4 μM) and eDHFR or eDHFR derivatives (4 μM) were incubated with **1** (10 μM) and acyl donor **2**–**6** (100 μM) in 50 mM Tris-HCl (pH 7.5), 100 mM NaCl, 0.2 mM tris(2-carboxyethyl)phosphine (TCEP), 0.01% Triton X-100, 0.3 mM DTT, and 0.3 mM EDTA at 37 °C for 5 h. After trichloroacetic acid (TCA) precipitation, the proteins were used for LC–MS/MS sample preparation.

Recombinant nucleosomes (33 ng/mL for DNA concentration, approximately 0.35 μM) and eDHFR or eDHFR derivatives (2 μM) were incubated with **1** (5 μM) and acyl donors **2**–**6** (100 μM) in 50 mM Tris-HCl (pH 7.5), 100 mM NaCl, 0.2 mM DTT, 0.2 mM TCEP, and 0.2 mM EDTA at 37 °C for 5 h. For LC–MS/MS analysis, proteins were precipitated by TCA precipitation, and DNA in nucleosomes was digested with DNase I (TaKaRa, 2270A). After acetone precipitation, the proteins were used for LC–MS/MS sample preparation. For immunoblotting, the reaction mixture was boiled with SDS sample buffer and analyzed by CBB staining and Western blotting using anti-acetyl or anti-butyryl lysine antibody. To detect acylation containing an azide or alkyne group, the reaction mixture was denatured by boiling with SDS sample buffer without DTT. Tris((1-benzyl-1*H*-1,2,3-triazol-4-yl)methyl)amine (TBTA, 500 μM), CuSO_4_ (250 μM), TAMRA-azide (100 μM, Sigma-Aldrich, 760757), or TAMRA-alkyne (click chemistry tools, TA-108), and sodium ascorbate (2 mM) were added to the mixture, and the mixture was incubated at 25 °C for 1 h. The reaction was quenched by adding cold acetone (80%, final concentration), and the mixture was stored at −20 °C. After centrifugation (15 000 rpm for 20 min at 4 °C), the supernatant was removed and the protein pellet was dissolved in SDS sample buffer with DTT. The samples were separated by SDS-PAGE, and the fluorescence was directly detected by ImageQuant LAS4000 (GE Healthcare).

### Sample Preparation for LC–MS/MS Analysis

The protein pellets were dissolved in Milli-Q water (MQ), added the same volume of 200 mM aqueous ammonium bicarbonate (NH_4_HCO_3_ aq) and twice the volume of 25% propionic anhydride solution (methanol/propionic anhydride, 3:1 (v/v)), and then the pH was adjusted to 8–9 by adding ammonia solution. After the propionylation reaction at 25 °C for 30 min, the solvents were removed with a Speed-Vac evaporator. The samples were resuspended in 50 mM NH_4_HCO_3_ aq with 0.1% ProteaseMAX (Promega, V2072) and digested with 10 or 20 ng/μL Trypsin Gold (Promega, V5280), 10 ng/μL Glu-C (Promega, V1651), and/or 2 ng/μL Asp-N (Promega, V1621) in 50 mM NH_4_HCO_3_ aq with 0.02 or 0.03% ProteaseMAX at 37 °C for 3 h to overnight. The digestion enzymes for each peptide are indicated in Tables S2–6. Then 5% aqueous formic acid (v/v) was added, and the solvents were removed with a Speed-Vac evaporator to obtain dried digested samples, which were dissolved in 0.1% aqueous formic acid (v/v). After centrifugation (15 000 rpm, 10 min), the supernatant was used for LC–MS/MS analysis.

### LC–MS/MS Analysis and Quantification of the Stoichiometry of Acetylation

LC–MS/MS analyses were conducted as described previously,^11^ with some modifications. LC was carried out as follows: 3C18-CL-120 column (0.5 mm inner diameter ×100 mm) with a linear gradient of 2–51.5% acetonitrile with 0.1% formic acid (v/v) versus water with 0.1% formic acid (v/v) over 9 min for MDM2, or with a linear gradient of 2–35% acetonitrile with 0.1% formic acid (v/v) versus water with 0.1% formic acid (v/v) over 6 min for histones, at 40 °C with a flow rate of 20 μL/min after 1 min equilibration. Targeted precursor ions and collision energies are described in Tables S2–6. Data analysis was carried out using PeakView software (AB Sciex, version 1.2.0.3) as described previously,^11^ and the average values obtained from duplicated measurements were adopted as the results for each assay. The stoichiometry of acetylated lysines was calculated from the extracted ion chromatogram as a percentage: the total peak area for acetylated peptides out of the combined total peak areas for both acetylated and propionylated peptides. Selected fragment ions are also shown in Tables S2–6. Two independent experiments were conducted. When the acetylation fragments were not detected in either experiment, the acetylation yield was considered “not detected”.

### Cell Culture

HEK293T and HeLa S3 cells were grown in Dulbecco’s modified Eagle’s medium (DMEM; Gibco, 12430) with 10% fetal bovine serum (Biowest, S1810), 1× GlutaMAX (Gibco), 100 U/mL penicillin, and 100 μg/mL streptomycin (Wako) at 37 °C in an atmosphere with 5% CO_2_.

### Localization of eDHFR or eDHFR Derivatives in Living Cells

To examine the subcellular distribution of eDHFR or eDHFR derivatives, TMP-TAMRA was imaged by fluorescent microscopy. HEK293T cells were cultured in poly-d-lysine-coated glass-bottom dishes and transfected with pcDNA5/TO-eDHFR/eDHFR derivatives-FLAG plasmids by Lipofectamine LTX reagent and Plus reagent (Invitrogen, 15338100) according to the manufacturer’s instructions. After 24 h, the cells were treated with 330 nM nocodazole for 4 h, and 10 μM TMP-TAMRA and 330 nM nocodazole for 1 h. After washing twice with growth medium, the cells were incubated with 330 nM nocodazole and 1 μg/mL Hoechst 33342 for 30 min. Images were captured using a fluorescent microscope (Leica, DMi8).

### Acetylation Assay in Living Cells

HEK293T cells were transfected with pcDNA5/TO-eDHFR/PLIED-FLAG or pcDNA5/TO-LANA-eDHFR/eDHFR-LANA plasmids by Lipofectamine LTX reagent and Plus reagent according to the manufacturer’s instructions. After 24 h, the cells were treated with **1** and **2** in Opti-MEM (gibco) at 37 °C. For immunoblotting of whole cell extracts, the cells were harvested and lysed with CRB buffer (50 mM Tris-HCl (pH 7.5), 300 mM NaCl, 0.3% Triton X-100) supplemented with 0.1 U/mL benzonase nuclease (Millipore, 70664), 2 mM MgCl_2_, 1× protease inhibitor cocktail (Sigma-Aldrich, P2714), and 1 mM PMSF on ice for 30 min. After centrifugation (15 000 rpm, 20 min), the supernatant was used for Western blotting with indicated antibodies or SDS-PAGE followed by Oriole or CBB staining. For immunoblotting of histones, the cells were harvested and the histones were acid extracted. The histones were dissolved in MQ, and pH was adjusted by adding 1/10 volume of 1 M Tris-HCl (pH 7.5). To equalize the concentrations of the histones, the samples were analyzed by SDS-PAGE followed by Oriole staining, and the signals were detected by BioDoc-It Imaging System (UVP) and measured by ImageJ. After equalizing the histone concentration, the histones were analyzed by Western blotting with indicated antibodies. Antibodies are listed in Table S7. For LC–MS/MS analysis, histones were isolated with Histone Purification Kit (Active Motif) according to the manufacturer’s instructions, precipitated by perchloric acid, and used for LC–MS/MS sample preparation.

### siRNA Treatment

HEK293T cells were transfected with control siRNA (SantaCruz, sc-37007) or RNF20 siGENOME SMARTpool siRNA (Dharmacon) by Lipofectamine RNAiMax transfection reagent (Invitrogen, 13778075) according to the manufacturer’s instructions. After 10 or 24 h, the cells were harvested and analyzed as in Acetylation Assay in Living Cells.

### ChIP (Chromatin Immunoprecipitation) Assay

HEK293T cells were treated as described in Acetylation Assay in Living Cells. After the acetylation reaction, Opti-MEM containing **1** and **2** was replaced with growth medium. To fix the cells, 27 μL of 37% formaldehyde was added per 1 mL of medium. After incubation at room temperature for 15 min, 80 μL of 1.25 M glycine in PBS was added per 1 mL of medium to quench the fixation reaction, and the cells were harvested and washed with PBS twice. The cells were suspended in SDS lysis buffer (50 mM Tris-HCl (pH 8.1), 1% SDS, 10 mM EDTA, protease inhibitor cocktail), incubated on ice for 10 min, and sonicated with BioRaptor II (BM Equipment Co). The supernatants were diluted 10-fold with ChIP dilution buffer (16.7 mM Tris-HCl (pH 8.1) 0.01% SDS, 1.1% Triton X-100, 1.2 mM EDTA, 167 mM NaCl). The DNA concentrations were determined with Nanodrop lite (Thermo) and then adjusted to 100 ng/μL. For H2BK120 and normal rabbit IgG immunoprecipitations (IPs), 300 μL of extract (100 ng/μL for DNA) was incubated with 1 μg antibodies for 30 min on ice. For H3K79me2, H3, normal rabbit IgG, H2BK120ac, and normal mouse IgG IPs, 150 μL of extract was incubated with 1 μg antibodies for 30 min on ice. After adding Dynabeads Protein G (20 μL, Veritas), the extract-antibody mixtures were incubated at 4 °C for 2 h with rotating. After washing in LS buffer (20 mM Tris-HCl (pH 8.1), 150 mM NaCl, 0.1% SDS, 1% Triton X-100, 2 mM EDTA), HS buffer (20 mM Tris-HCl (pH 8.1), 500 mM NaCl, 0.1% SDS, 1% Triton X-100, 2 mM EDTA), LiCl buffer (10 mM Tris-HCl (pH 8.1), 0.25 M LiCl, 1% NP-40, 1% sodium deoxycholate, 1 mM EDTA), and TE buffer (10 mM Tris-HCl (pH 8), 1 mM EDTA), the coimmunoprecipitated DNA was extracted at room temperature for 15 min with elution buffer (100 mM NaHCO_3_, 1% SDS, 10 mM DTT). The supernatant was collected, incubated at 65 °C overnight, and then treated with Proteinase K (Takara, 9034) at 45 °C for 1–3 h. For input sample, 150 μL of extract (100 ng/ μL for DNA) was incubated with 200 mM NaCl at 65 °C overnight and treated with Proteinase K at 45 °C for 1–3 h. DNA was purified with PCR Clean-Up Mini Kit (Favorgen) before performing PCR. Purified DNA was analyzed by quantitative PCR using LightCycler 480 system (Roche) with SYBR Green I Master (Roche). The amount of coimmunoprecipitated DNA was divided by the amount of total DNA of input to calculate IP (%) of each antibody. Then the IP (%) for each antibody (%) was calculated by subtracting IgG IP (%) from the total IP (%). Primers used in ChIP assays are listed in Table S8.

### Acylation Assay in Living Cells

HEK293T cells were transfected with pcDNA5/TO-PLIED-L52-FLAG (1/16) and empty (15/16) plasmid mixture by Lipofectamine LTX reagent and Plus reagent according to the manufacturer’s instructions. After 24 h, the cells were treated with **1** and **3**, **4**, or **5** in Opti-MEM at 37 °C for 5 h. The cells were harvested and lysed with CRB buffer (50 mM Tris-HCl (pH 7.5), 300 mM NaCl, 0.3% Triton X-100) supplemented with 0.1 U/mL benzonase nuclease, 2 mM MgCl_2_, and 1 mM PMSF on ice for 30 min. After centrifugation (15 000 rpm, 20 min), the supernatant was used for Western blotting to detect butyrylation. To detect acylation containing azide or alkyne, the supernatant (0.5 mg/mL for total protein concentration) was denatured by boiling with SDS sample buffer without DTT. Tris((1-benzyl-1*H*-1,2,3-triazol-4-yl)methyl)amine (TBTA, 500 μM), CuSO_4_ (250 μM), TAMRA-azide (100 μM, Sigma-Aldrich, 760757) or TAMRA-alkyne (click chemistry tools, TA-108), and sodium ascorbate (2 mM) were added to the mixture, and the mixture was incubated at 25 °C for 1 h. The reaction was quenched by adding cold acetone (80%, final concentration), and the mixture was stored at −20 °C. After centrifugation (15 000 rpm for 20 min at 4 °C), the supernatant was removed and the protein pellet was dissolved in SDS sample buffer with DTT. The samples were separated by SDS-PAGE, and the fluorescence was directly detected by ImageQuant LAS4000 (GE Healthcare).

### Imaging of Acylated Histones in Cells

HeLaS3 cells were transfected with pcDNA5/TO-PLIED-L52-FLAG (1/4), pEGFP-N1 (1/20) and empty plasmid mixture by Lipofectamine 3000 transfection reagent (Invitrogen, L3000015) according to manufacturer’s instructions. After 22 h, the cells were treated with **1** and **5** in Opti-MEM at 37 °C for 5 h. The cells were washed with PBS, followed by fixation with 4% paraformaldehyde in PBS at room temperature for 10 min. The fixed cells were washed with PBS and treated with 0.5% Triton X-100 in PBS and then 3% bovine serum albumin (BSA; Sigma, 10735086001) in PBS. Then Cu(I)-catalyzed azide–alkyne cycloaddition was conducted with 50 μM TAMRA-azide (Sigma-Aldrich, 760757), 200 μM tris-hydroxypropyltriazolylmethylamine (THPTA), 50 μM CuSO_4_, and 2.5 mM sodium ascorbate in PBS at 25 °C for 1 h. The cells were washed with PBS five times and the chromosomal DNA were visualized by 1 μg/mL DAPI staining. The cells were washed with PBS and imaged by fluorescent microscopy (DMi8; Leica).

## Supporting Information Available

Synthesis, expression plasmids, antibodies; Figure S1, related to Figure 2; Figure S2, related to Figure 3; Figure S3, related to Figure 4; Figure S4, related to Figure 4; Figure S5, related to Figure 5; Figure S6, related to Figures 2–4; Table S1. Plasmids for protein expression; Table S2. LC–MS/MS parameters for MDM2; Table S3. LC–MS/MS parameters for histone H2A; Table S4. LC–MS/MS parameters for histone H2B; Table S5. LC–MS/MS parameters for histone H3; Table S6. LC–MS/MS parameters for histone H4; Table S7. Antibodies for Western blotting or ChIP assay; Table S8. Primers for real-time PCR (PDF)
Transparent Peer Review report available (PDF)
