# Supplementary material for: Designer Adaptor Proteins for Functional Conversion of Peptides to Small-Molecule Ligands toward In-Cell Catalytic Protein Modification
Source: ACS Cent Sci. 2023 Oct 25;9(11):2115–28. doi: 10.1021/acscentsci.3c00930 (PMC10683481; doi:10.1021/acscentsci.3c00930)
Supplement: Supplementary file 1 — oc3c00930_si_001.pdf [file oc3c00930_si_001.pdf]

Supporting Information for

Designer adaptor proteins for functional conversion of peptides to small-molecule ligands toward in-cell catalytic protein modification

Akiko Fujimura[1], Hisashi Ishida[2], Tamiko Nozaki[1], Shuhei Terada[1], Yuto Azumaya[1], Tadashi Ishiguro[1], Yugo R. Kamimura[1], Tomoya Kujirai[3], Hitoshi Kurumizaka[3], Hidetoshi Kono[2], Kenzo Yamatsugu[1], Shigehiro A. Kawashima[1,*], & Motomu Kanai[1,*]

[1]Graduate School of Pharmaceutical Sciences, The University of Tokyo, Tokyo 113-0033, Japan, [2]Institute for Quantum Life Science, National Institutes for Quantum Science and Technology, Kyoto, 619-0215, Japan, [3]Institute for Quantitative Biosciences, The University of Tokyo, Tokyo 113-0032, Japan

*Corresponding Authors:

Motomu Kanai

kanai@mol.f.u-tokyo.ac.jp

Shigehiro A. Kawashima

skawashima@mol.f.u-tokyo.ac.jp

Table of contents

Synthesis	S3
Expression plasmids	S5
Antibodies	S9
References	S10
Figure S1-S4	S11
Table S1-S8	S17

General

NMR spectra were recorded on JEOL ECX500 spectrometer, operating at 500 MHz for 1H NMR or JEOL ECS400 spectrometer, operating at 400 MHz for 1H NMR. Chemical shifts were reported in ppm on the δ scale relative to residual CD_3OD (δ = 3.31 for 1H NMR) and $(CD_3)_2CO$ (δ = 2.04 for 1H NMR) as an internal reference, respectively. Preparative HPLC was conducted by using a JASCO HPLC system equipped with a UV-2075 spectrometer, PU-2086 pumps, a DG-2080-53 degasser, and an MX-2080-32 mixer. High resolution mass spectra were recorded using a Bruker microTOF ESI-TOF mass spectrometer.

Synthesis of TMP-conjugated TAMRA

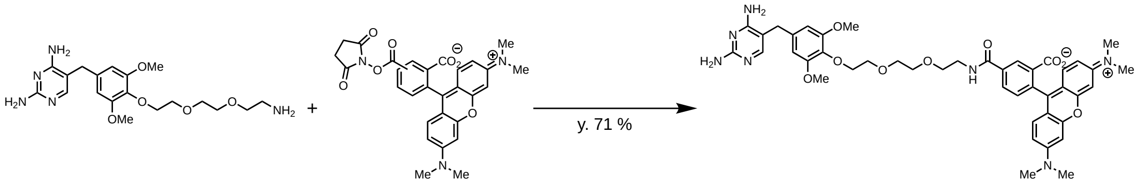

To a stirred solution of 5-(4-(2-(2-(2-aminoethoxy)ethoxy)ethoxy)-3,5-dimethoxybenzyl)pyrimidine-2,4-diamine[1] (2.7 mg, 6.63 µmol) and NHS-tetramethylrhodamine (5,6-isomer, 4.2 mg, 87.96 µmol) in DMF (200 µL), 0.1 M $NaHCO_3$ aq. was added. The mixture was stirred at room temperature. After 25 min, 50 µL H_2O was added and the mixture was aged for a further 90 min. The mixture was concentrated and purified with preparative HPLC (2 % acetonitrile for 5 min, followed by a linear gradient of 2-100 % acetonitrile over 40 min in 0.1% TFA aqueous solution, YMC-Triart C18, 254 nm) to afford TMP-conjugated TAMRA (5-isomer was separated., 3.87 mg, 4.72 µmol, 71 % yield) as red powder. ^{1}H NMR (CD_3OD, 500 MHz) δ 8.74 (d, J = 1.7 Hz, 1H), 8.24 (dd, J = 1.7 Hz, J = 7.5 Hz, 1H), 7.48 (d, J = 8.0 Hz, 1H), 7.11 (m, 3H), 7.02 (dd, J = 2.3 Hz, J = 9.2 Hz, 2H), 6.96 (d, J = 2.9 Hz, 2H), 6.53 (s, 2H), 4.07 (m, 2H), 3.76-3.72 (m, 16H), 3.61 (s, 1H), (12H from tetramethyl moiety seemed to be overlapped with CHD_2OD.);
ESI-HRMS m/z calcd for $C_{44}H_{49}N_7O_9$ $[M+H]^+$: 820.3665; Found 820.3648

Synthesis of 6

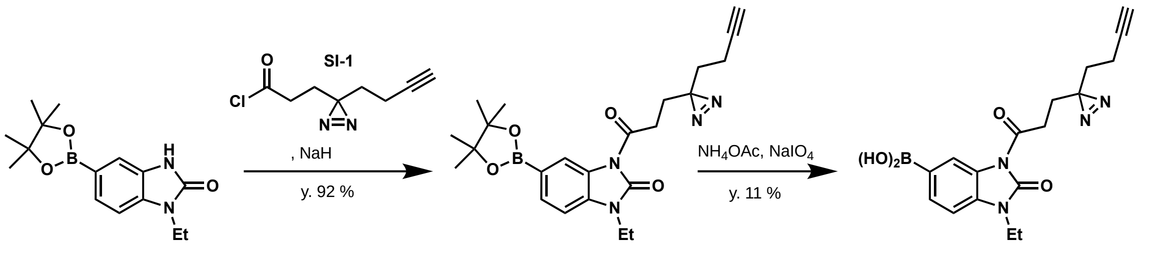

To a stirred solution of **SI-1** (9.5 mg, 57 μmol, 4.7 eq)[2] in 300 μL CH_2Cl_2 was charged 0.2 μL DMF followed by oxalyl chloride (7.4 μL, 85 μmol, 7.0 eq). After 45 min, the reaction was concentrated on the rotovap and co-evaporated with 100 μL CH_2Cl_2.

To a stirred 0.2 M solution of **SI-2** (3.5 mg, 12 μmol)[3] in DMF, was charged NaH (55% suspension, 0.64 mg, 15 μmol, 1.2 eq). After 5 min, the crude acid chloride was charged as a solution in 50 μL CH_2Cl_2. After 15 min, the reaction was quenched to a stirred mixture of 2 mL EtOAc and 1 mL pH 4 buffer. The aqueous material was discarded, and the organic material was washed twice with brine, dried ($MgSO_4$), filtered, and concentrated on the rotovap. The crude material was purified with preparative HPLC (30 % acetonitrile for 5 min, followed by a linear gradient of 30-100 % acetonitrile over 70 min in 0.1% TFA aqueous solution, YMC-Triart C18, 254 nm) to afford the intermediate boronic ester **SI-3** (5.3 mg, 11 μmol, 92% yield) as a white solid.

To a stirred suspension of the intermediate boronic ester **SI-3** (3.2 mg, 7.3 μmol) in 200 μL 2 : 1 v/v acetone : H_2O was charged NH_4OAc (2M solution in H_2O, 7.3 μL, 15 μmol, 2 eq) followed by $NaIO_4$ (4.7 mg, 22 μmol, 3 eq), and the mixture aged for overnight at 35 °C. Most of the acetone was removed on the rotovap. The mixture was extracted with 1 mL EtOAc and 1 mL H_2O and the aqueous material was discarded. The organic material was washed with brine, dried ($MgSO_4$), and concentrated on the rotovap. Purification by preparative HPLC (10 % acetonitrile for 5 min, followed by a linear gradient of 10-80 % acetonitrile over 100 min in 0.1% TFA aqueous solution, YMC-Triart PFP, 254 nm) afforded **6** (0.28 mg, 0.79 μmol, 11% yield) as a white solid.

^{1}H NMR (($(CH_3)_2CO$, 400 MHz) δ 8.62 (s, 1H), 7.99 (bs, 1H), 7.80 (d, *J* = 7.6 Hz, 1H), 7.17 (d, *J* = 7.6 Hz, 1H), 3.93 (q, *J* = 6.3 Hz, 2H), 3.05 (t, *J* = 7.6 Hz, 2H), 2.39 (br, 1H), 1.73 (t, *J* = 7.2 Hz, 2H), 1.30 (t, *J* = 7.2 Hz, 7H);

ESI-HRMS *m/z* calcd for $C_{17}H_{19}BN_4O_4$ $[M+Na]^+$: 377.1392; Found 377.1398

Expression plasmids

Plasmids were constructed from pGEX-6P-2-eDHFR-GFP plasmid[1] by ligating DNA fragments using DNA Ligation kit (TaKaRa) or site-directed mutagenesis using Prime STAR MAX (TaKaRa). All plasmids used in this paper are listed in Table S1. List of protein sequences is shown below. Texts in red indicate the affinity tags that were cleaved during purification.

> GST-hMDM2(17-125) from pGEX-6P-2-hMDM2(17-125)

MSPILGYWKIKGLVQPTRLLLEYLEEKYEEHLYERDEGDKWRNKKFELGLEFP
NLPYYIDGDVKLTQSMAIIRYIADKHNMLGGCPKERAEISMLEGAVLDIRYGVSR
IAYSKDFETLKVDFLSKLPEMLKMFEDRLCHKTYLNGDHVTHPDFMLYDALDV
VLYMDPMCLDAFPKLVCFKKRIEAIPQIDKYLKSSKYIAWPLQGWQATFGGGD
HPPKSDLEVLFQGPLGSSQIPASEQETLVRPKPLLLKLLKSVGAQKDTYTMKEV
LFYLGQYIMTKRLYDEKQQHIVYCSNDLLGDLFGVPSFSVKEHRKIYTMIYRNL
VVVNQQESSDSGTSVSEN

> GST-eDHFR(K32R) from pGEX-6P-2-eDHFR(K32R)

MSPILGYWKIKGLVQPTRLLLEYLEEKYEEHLYERDEGDKWRNKKFELGLEFP
NLPYYIDGDVKLTQSMAIIRYIADKHNMLGGCPKERAEISMLEGAVLDIRYGVSR
IAYSKDFETLKVDFLSKLPEMLKMFEDRLCHKTYLNGDHVTHPDFMLYDALDV
VLYMDPMCLDAFPKLVCFKKRIEAIPQIDKYLKSSKYIAWPLQGWQATFGGGD
HPPKSDLEVLFQGPLGSMISLIAALAVDRVIGMENAMPWNLPADLAWFRRNTL
NKPVIMGRHTWESIGRPLPGRKNIILSSQPGTDDRVTWVKSVDEAIAACGDVP
EIMVIGGGRVYEQFLPKAQKLYLTHIDAEVEGDTHFPDYEPDDWESVFSEFHD
ADAQNSHSYCFEILERR

> GST-eDHFR(K32R)-MBP1 from pGEX-6P-2-eDHFR(K32R)-MBP1

MSPILGYWKIKGLVQPTRLLLEYLEEKYEEHLYERDEGDKWRNKKFELGLEFP
NLPYYIDGDVKLTQSMAIIRYIADKHNMLGGCPKERAEISMLEGAVLDIRYGVSR
IAYSKDFETLKVDFLSKLPEMLKMFEDRLCHKTYLNGDHVTHPDFMLYDALDV
VLYMDPMCLDAFPKLVCFKKRIEAIPQIDKYLKSSKYIAWPLQGWQATFGGGD
HPPKSDLEVLFQGPLGSMISLIAALAVDRVIGMENAMPWNLPADLAWFRRNTL
NKPVIMGRHTWESIGRPLPGRKNIILSSQPGTDDRVTWVKSVDEAIAACGDVP
EIMVIGGGRVYEQFLPKAQKLYLTHIDAEVEGDTHFPDYEPDDWESVFSEFHD
ADAQNSHSYCFEILERRETFEHWWSQLLS

> GST-MBP1-eDHFR(K32R) from pGEX-6P-2-MBP1-eDHFR(K32R)

MSPILGYWKIKGLVQPTRLLLEYLEEKYEEHLYERDEGDKWRNKKFELGLEFP
NLPYYIDGDVKLTQSMAIIRYIADKHNMLGGCPKERAEISMLEGAVLDIRYGVSR
IAYSKDFETLKVDFLSKLPEMLKMFEDRLCHKTYLNGDHVTHPDFMLYDALDV
VLYMDPMCLDAFPKLVCFKKRIEAIPQIDKYLKSSKYIAWPLQGWQATFGGGD
HPPKSDLEVLFQGPLGSETFEHWWSQLLSISLIAALAVDRVIGMENAMPWNLP
ADLAWFRRNTLNKPVIMGRHTWESIGRPLPGRKNIILSSQPGTDDRVTWVKSV
DEAIAACGDVPEIMVIGGGRVYEQFLPKAQKLYLTHIDAEVEGDTHFPDYEPDD
WESVFSEFHDADAQNSHSYCFEILERR

> GST-PLIED-M1(K32R) from pGEX-6P-2-PLIED-M1(K32R)
MSPILGYWKIKGLVQPTRLLLEYLEEKYEEHLYERDEGDKWRNKKFELGLEFP
NLPYYIDGDVKLTQSMAIIRYIADKHNMLGGCPKERAEISMLEGAVLDIRYGVSR
IAYSKDFETLKVDFLSKLPEMLKMFEDRLCHKTYLNGDHVTHPDFMLYDALDV
VLYMDPMCLDAFPKLVCFKKRIEAIPQIDKYLKSSKYIAWPLQGWQATFGGGD
HPPKSDLEVLFQGPLGSMISLIAALAVDRVIGMENAMPWNLPADLAWFRRNTL
NKPVIMGRHTWESIGGGETFEHWWSQLLSGGGRKNIILSSQPGTDDRVTWVK
SVDEAIAACGDVPEIMVIGGGRVYEQFLPKAQKLYLTHIDAEVEGDTHFPDYEP
DDWESVFSEFHDADAQNSHSYCFEILERR

> GST-PLIED-M2(K32R) from pGEX-6P-2-PLIED-M2(K32R)
MSPILGYWKIKGLVQPTRLLLEYLEEKYEEHLYERDEGDKWRNKKFELGLEFP
NLPYYIDGDVKLTQSMAIIRYIADKHNMLGGCPKERAEISMLEGAVLDIRYGVSR
IAYSKDFETLKVDFLSKLPEMLKMFEDRLCHKTYLNGDHVTHPDFMLYDALDV
VLYMDPMCLDAFPKLVCFKKRIEAIPQIDKYLKSSKYIAWPLQGWQATFGGGD
HPPKSDLEVLFQGPLGSMISLIAALAVDRVIGMENAMPWNLPADLAWFRRNTL
NKPVIMGRHTWESIGGGRFMDYWEGLGGGRKNIILSSQPGTDDRVTWVKSV
DEAIAACGDVPEIMVIGGGRVYEQFLPKAQKLYLTHIDAEVEGDTHFPDYEPDD
WESVFSEFHDADAQNSHSYCFEILERR

> GST-PLIED-M3(K32R) from pGEX-6P-2-PLIED-M3(K32R)
MSPILGYWKIKGLVQPTRLLLEYLEEKYEEHLYERDEGDKWRNKKFELGLEFP
NLPYYIDGDVKLTQSMAIIRYIADKHNMLGGCPKERAEISMLEGAVLDIRYGVSR
IAYSKDFETLKVDFLSKLPEMLKMFEDRLCHKTYLNGDHVTHPDFMLYDALDV
VLYMDPMCLDAFPKLVCFKKRIEAIPQIDKYLKSSKYIAWPLQGWQATFGGGD
HPPKSDLEVLFQGPLGSMISLIAALAVDRVIGMENAMPWNLPADLAWFRRNTL
NKPVIMGRHTWESIGGGTSFAEYWNLLGGGRKNIILSSQPGTDDRVTWVKSV

DEAIAACGDVPEIMVIGGGRVYEQFLPKAQKLYLTHIDAEVEGDTHFPDYEPDD
WESVFSEFHDADAQNSHSYCFEILERR

> GST-LANA-eDHFR(K32R) from pGEX-6P-2-LANA-eDHFR(K32R)
MSPILGYWKIKGLVQPTRLLLEYLEEKYEEHLYERDEGDKWRNKKFELGLEFP
NLPYYIDGDVKLTQSMAIIRYIADKHNMLGGCPKERAEISMLEGAVLDIRYGVSR
IAYSKDFETLKVDFLSKLPEMLKMFEDRLCHKTYLNGDHVTHPDFMLYDALDV
VLYMDPMCLDAFPKLVCFKKRIEAIPQIDKYLKSSKYIAWPLQGWQATFGGGD
HPPKSDLEVLFQGPLGSMGMRLRSGRSTGISLIAALAVDRVIGMENAMPWNLP
ADLAWFRRNTLNKPVIMGRHTWESIGRPLPGRKNIILSSQPGTDDRVTWVKSV
DEAIAACGDVPEIMVIGGGRVYEQFLPKAQKLYLTHIDAEVEGDTHFPDYEPDD
WESVFSEFHDADAQNSHSYCFEILERR

> GST-eDHFR(K32R)-LANA from pGEX-6P-2-eDHFR(K32R)-LANA
MSPILGYWKIKGLVQPTRLLLEYLEEKYEEHLYERDEGDKWRNKKFELGLEFP
NLPYYIDGDVKLTQSMAIIRYIADKHNMLGGCPKERAEISMLEGAVLDIRYGVSR
IAYSKDFETLKVDFLSKLPEMLKMFEDRLCHKTYLNGDHVTHPDFMLYDALDV
VLYMDPMCLDAFPKLVCFKKRIEAIPQIDKYLKSSKYIAWPLQGWQATFGGGD
HPPKSDLEVLFQGPLGSMISLIAALAVDRVIGMENAMPWNLPADLAWFRRNTL
NKPVIMGRHTWESIGRPLPGRKNIILSSQPGTDDRVTWVKSVDEAIAACGDVP
EIMVIGGGRVYEQFLPKAQKLYLTHIDAEVEGDTHFPDYEPDDWESVFSEFHD
ADAQNSHSYCFEILERRGMRLRSGRSTG

> GST-PLIED-L23(K32R) from pGEX-6P-2-PLIED-L23(K32R)
MSPILGYWKIKGLVQPTRLLLEYLEEKYEEHLYERDEGDKWRNKKFELGLEFP
NLPYYIDGDVKLTQSMAIIRYIADKHNMLGGCPKERAEISMLEGAVLDIRYGVSR
IAYSKDFETLKVDFLSKLPEMLKMFEDRLCHKTYLNGDHVTHPDFMLYDALDV
VLYMDPMCLDAFPKLVCFKKRIEAIPQIDKYLKSSKYIAWPLQGWQATFGGGD
HPPKSDLEVLFQGPLGSMISLIAALAVDRVIGMENAMPWNGMRLRSGRSTGLP
ADLAWFRRNTLNKPVIMGRHTWESIGRPLPGRKNIILSSQPGTDDRVTWVKSV
DEAIAACGDVPEIMVIGGGRVYEQFLPKAQKLYLTHIDAEVEGDTHFPDYEPDD
WESVFSEFHDADAQNSHSYCFEILERR

> GST-PLIED-L36(K32R) from pGEX-6P-2-PLIED-L36(K32R)
MSPILGYWKIKGLVQPTRLLLEYLEEKYEEHLYERDEGDKWRNKKFELGLEFP
NLPYYIDGDVKLTQSMAIIRYIADKHNMLGGCPKERAEISMLEGAVLDIRYGVSR

IAYSKDFETLKVDFLSKLPEMLKMFEDRLCHKTYLNGDHVTHPDFMLYDALDV
VLYMDPMCLDAFPKLVCFKKRIEAIPQIDKYLKSSKYIAWPLQGWQATFGGGD
HPPKSDLEVLFQGPLGSMISLIAALAVDRVIGMENAMPWNLPADLAWFRRNTL
GMRLRSGRSTGNKPVIMGRHTWESIGRPLPGRKNIILSSQPGTDDRVTWVKS
VDEAIAACGDVPEIMVIGGGRVYEQFLPKAQKLYLTHIDAEVEGDTHFPDYEPD
DWESVFSEFHDADAQNSHSYCFEILERR

> GST-PLIED-L51(K32R) from pGEX-6P-2-PLIED-L51(K32R)
MSPILGYWKIKGLVQPTRLLLEYLEEKYEEHLYERDEGDKWRNKKFELGLEFP
NLPYYIDGDVKLTQSMAIIRYIADKHNMLGGCPKERAEISMLEGAVLDIRYGVSR
IAYSKDFETLKVDFLSKLPEMLKMFEDRLCHKTYLNGDHVTHPDFMLYDALDV
VLYMDPMCLDAFPKLVCFKKRIEAIPQIDKYLKSSKYIAWPLQGWQATFGGGD
HPPKSDLEVLFQGPLGSMISLIAALAVDRVIGMENAMPWNLPADLAWFRRNTL
NKPVIMGRHTWESIGGMRLRSGRSTGRPLPGRKNIILSSQPGTDDRVTWVKS
VDEAIAACGDVPEIMVIGGGRVYEQFLPKAQKLYLTHIDAEVEGDTHFPDYEPD
DWESVFSEFHDADAQNSHSYCFEILERR

> GST-PLIED-L52(K32R) from pGEX-6P-2-PLIED-LR52(K32R)
MSPILGYWKIKGLVQPTRLLLEYLEEKYEEHLYERDEGDKWRNKKFELGLEFP
NLPYYIDGDVKLTQSMAIIRYIADKHNMLGGCPKERAEISMLEGAVLDIRYGVSR
IAYSKDFETLKVDFLSKLPEMLKMFEDRLCHKTYLNGDHVTHPDFMLYDALDV
VLYMDPMCLDAFPKLVCFKKRIEAIPQIDKYLKSSKYIAWPLQGWQATFGGGD
HPPKSDLEVLFQGPLGSMISLIAALAVDRVIGMENAMPWNLPADLAWFRRNTL
NKPVIMGRHTWESIGRGMRLRSGRSTGPLPGRKNIILSSQPGTDDRVTWVKS
VDEAIAACGDVPEIMVIGGGRVYEQFLPKAQKLYLTHIDAEVEGDTHFPDYEPD
DWESVFSEFHDADAQNSHSYCFEILERR

> eDHFR(K32R)-FLAG from pcDNA5/TO-eDHFR(K32R)-FLAG
MISLIAALAVDRVIGMENAMPWNLPADLAWFRRNTLNKPVIMGRHTWESIGRP
LPGRKNIILSSQPGTDDRVTWVKSVDEAIAACGDVPEIMVIGGGRVYEQFLPKA
QKLYLTHIDAEVEGDTHFPDYEPDDWESVFSEFHDADAQNSHSYCFEILERRG
DYKDDDDK

> LANA-eDHFR(K32R) from pcDNA5/TO-LANA-eDHFR(K32R)
MGMRLRSGRSTGISLIAALAVDRVIGMENAMPWNLPADLAWFRRNTLNKPVIM
GRHTWESIGRPLPGRKNIILSSQPGTDDRVTWVKSVDEAIAACGDVPEIMVIG

GGRVYEQFLPKAQKLYLTHIDAEVEGDTHFPDYEPDDWESVFSEFHDADAQN
SHSYCFEILERR

> eDHFR(K32R)-LANA from pcDNA5/TO-eDHFR(K32R)-LANA
MISLIAALAVDRVIGMENAMPWNLPADLAWFRRNTLNKPVIMGRHTWESIGRP
LPGRKNIILSSQPGTDDRVTWVKSVDEAIAACGDVPEIMVIGGGRVYEQFLPKA
QKLYLTHIDAEVEGDTHFPDYEPDDWESVFSEFHDADAQNSHSYCFEILERRG
MRLRSGRSTG

> PLIED-L23(K32R)-FLAG from pcDNA5/TO-PLIED-L23(K32R)-FLAG
MISLIAALAVDRVIGMENAMPWNGMRLRSGRSTGLPADLAWFRRNTLNKPVIM
GRHTWESIGRPLPGRKNIILSSQPGTDDRVTWVKSVDEAIAACGDVPEIMVIG
GGRVYEQFLPKAQKLYLTHIDAEVEGDTHFPDYEPDDWESVFSEFHDADAQN
SHSYCFEILERRGDYKDDDDK

> PLIED-L36(K32R)-FLAG from pcDNA5/TO-PLIED-L36(K32R)-FLAG
MISLIAALAVDRVIGMENAMPWNLPADLAWFRRNTLGMRLRSGRSTGNKPVIM
GRHTWESIGRPLPGRKNIILSSQPGTDDRVTWVKSVDEAIAACGDVPEIMVIG
GGRVYEQFLPKAQKLYLTHIDAEVEGDTHFPDYEPDDWESVFSEFHDADAQN
SHSYCFEILERRGDYKDDDDK

> PLIED-L51(K32R)-FLAG from pcDNA5/TO-PLIED-L51(K32R)-FLAG
MISLIAALAVDRVIGMENAMPWNLPADLAWFRRNTLNKPVIMGRHTWESIGGM
RLRSGRSTGRPLPGRKNIILSSQPGTDDRVTWVKSVDEAIAACGDVPEIMVIG
GGRVYEQFLPKAQKLYLTHIDAEVEGDTHFPDYEPDDWESVFSEFHDADAQN
SHSYCFEILERRGDYKDDDDK

> PLIED-L52(K32R)-FLAG from pcDNA5/TO-PLIED-L52(K32R)-FLAG
MISLIAALAVDRVIGMENAMPWNLPADLAWFRRNTLNKPVIMGRHTWESIGRG
MRLRSGRSTGPLPGRKNIILSSQPGTDDRVTWVKSVDEAIAACGDVPEIMVIG
GGRVYEQFLPKAQKLYLTHIDAEVEGDTHFPDYEPDDWESVFSEFHDADAQN
SHSYCFEILERRGDYKDDDDK

Antibodies

All antibodies used in this paper are listed in Table S7.

References

(1) Hamajima, W.; Fujimura, A.; Fujiwara, Y.; Yamatsugu, K.; Kawashima, S. A.; Kanai, M. Site-Selective Synthetic Acylation of a Target Protein in Living Cells Promoted by a Chemical Catalyst/Donor System. *ACS Chem Biol* **2019**, *14* (6), 1102-1109. DOI: 10.1021/acschembio.9b00102.

(2) Li, Z. Q.; Hao, P. L.; Li, L.; Tan, C. Y. J.; Cheng, X. M.; Chen, G. Y. J.; Sze, S. K.; Shen, H. M.; Yao, S. Q. Design and Synthesis of Minimalist Terminal Alkyne-Containing Diazirine Photo-Crosslinkers and Their Incorporation into Kinase Inhibitors for Cell- and Tissue-Based Proteome Profiling. *Angew Chem Int Edit* **2013**, *52* (33), 8551-8556. DOI: 10.1002/anie.201300683.

(3) Adamson, C.; Kajino, H.; Kawashima, S. A.; Yamatsugu, K.; Kanai, M. Live-Cell Protein Modification by Boronate-Assisted Hydroxamic Acid Catalysis. *J Am Chem Soc* **2021**, *143* (37), 14976-14980. DOI: 10.1021/jacs.1c07060.

(4) Fujiwara, Y.; Yamanashi, Y.; Fujimura, A.; Sato, Y.; Kujirai, T.; Kurumizaka, H.; Kimura, H.; Yamatsugu, K.; Kawashima, S. A.; Kanai, M. Live-cell epigenome manipulation by synthetic histone acetylation catalyst system. *Proc Natl Acad Sci U S A* **2021**, *118* (4). DOI: 10.1073/pnas.2019554118.

(5) Shema-Yaacoby, E.; Nikolov, M.; Haj-Yahya, M.; Siman, P.; Allemand, E.; Yamaguchi, Y.; Muchardt, C.; Urlaub, H.; Brik, A.; Oren, M.; et al. Systematic identification of proteins binding to chromatin-embedded ubiquitylated H2B reveals recruitment of SWI/SNF to regulate transcription. *Cell Rep* **2013**, *4* (3), 601-608. DOI: 10.1016/j.celrep.2013.07.014. David, Y.; Vila-Perello, M.; Verma, S.; Muir, T. W. Chemical tagging and customizing of cellular chromatin states using ultrafast trans-splicing inteins. *Nat Chem* **2015**, *7* (5), 394-402. DOI: 10.1038/nchem.2224.

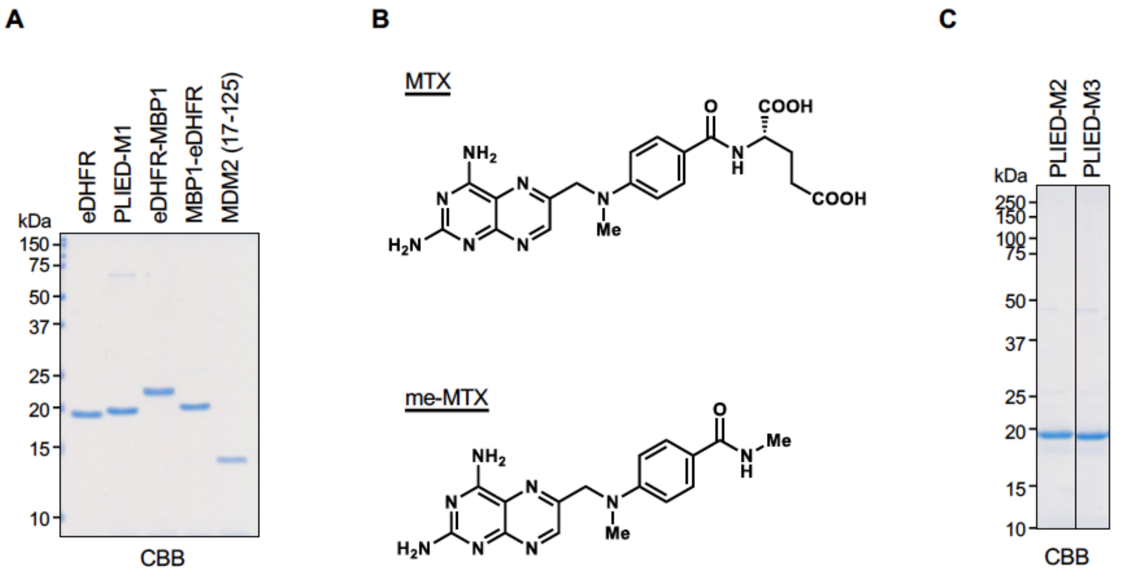

Figure S1, related to Figure 2.

A, Purified proteins of eDHFR derivatives used in Figure 2C. Purified eDHFR derivatives proteins were analyzed by SDS-PAGE and visualized by CBB staining. **B**, Chemical structure of MTX and me-MTX. **C**, Purified proteins of eDHFR derivatives used in Figure 2H. Purified PLIED proteins were analyzed as in **A**.

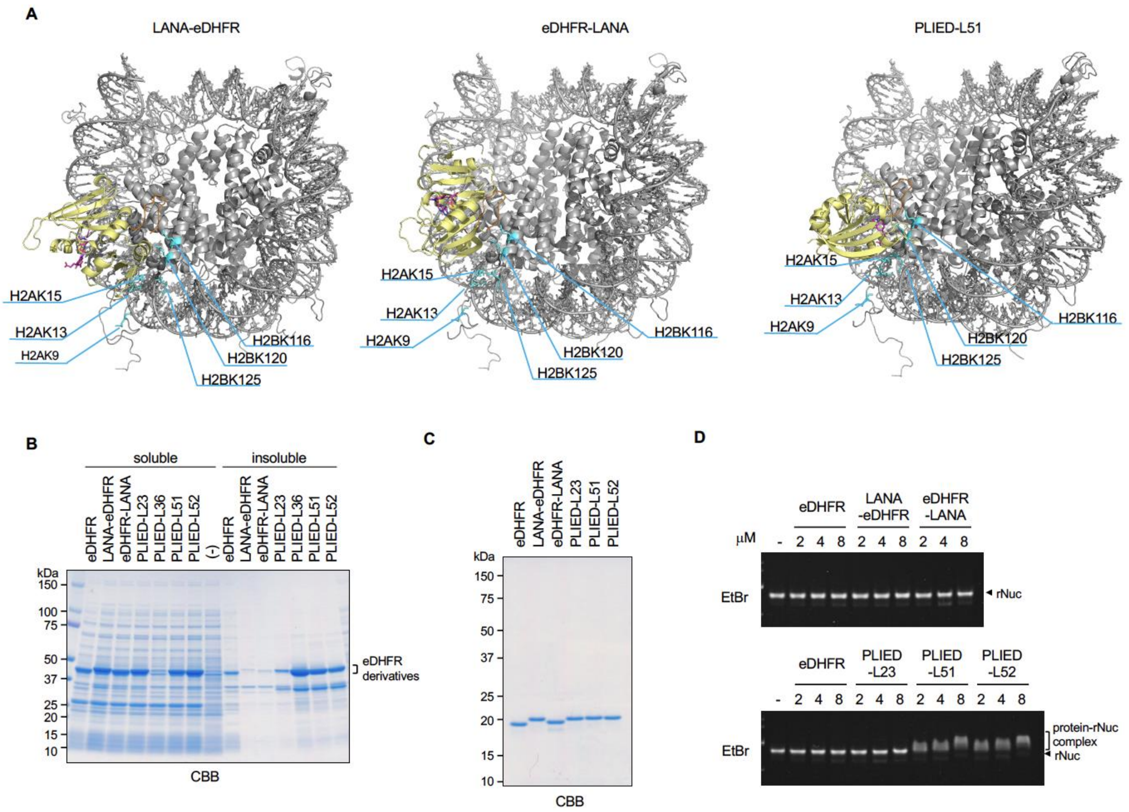

Figure S2, related to Figure 3.

A, Modelled structures of LANA-inserted eDHFR-nucleosome complex. The LANA (5-15) was implanted at the N-terminus (left), the C-terminus (middle), or between G51 and R52 (right) of eDHFR. eDHFR, LANA, MTX and lysines are shown in pale yellow, orange, magenta and cyan with labels, respectively. **B**, The eDHFR derivatives purification. The eDHFR derivatives-expressed *E. coli* BL21 C^+ cells were resuspended in solubilization buffer and sonicated. After centrifugation, the supernatant and the pellet were analyzed by SDS-PAGE as soluble and insoluble fractions, respectively, and proteins were visualized by CBB staining. **C**, Purified proteins of eDHFR derivatives. Purified proteins (50 μM) were analyzed by SDS-PAGE and visualized by CBB staining. **D**, Electrophoretic mobility shift assay of PLIED-bound nucleosomes. Recombinant nucleosomes (0.2 μM) were incubated with the indicated proteins (2, 4, and 8 μM). The samples were analyzed by 6% non-denaturing PAGE in 0.5× TBE buffer, and DNA was visualized by ethidium bromide (EtBr) staining. The positions of recombinant nucleosomes (rNuc) are shown. Representative data of two independent experiments are shown.

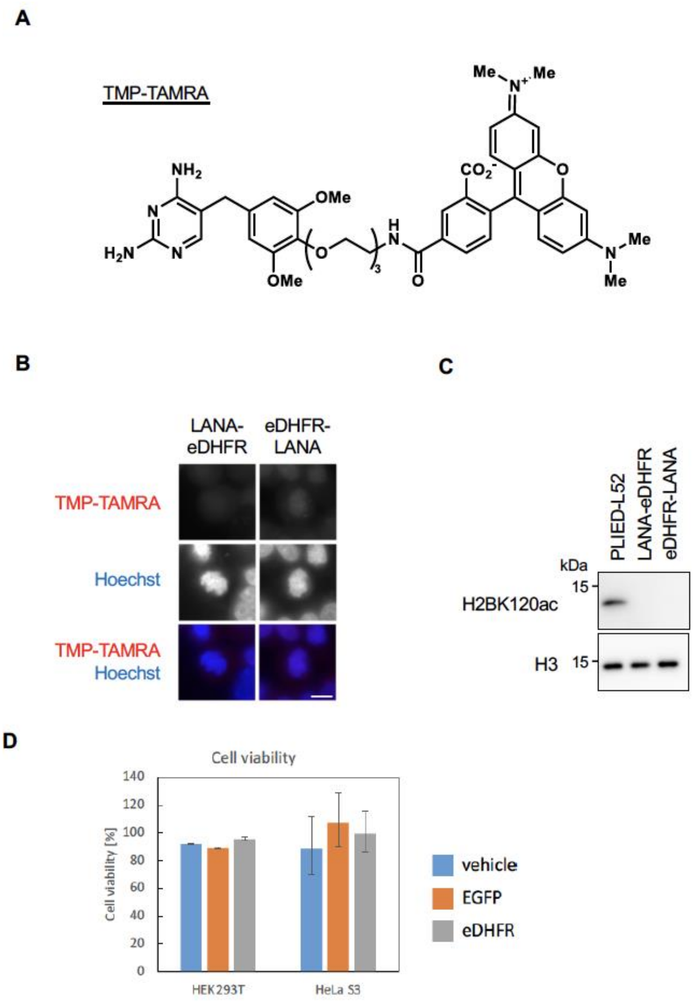

Figure S3, related to Figure 4.

A, Chemical structure of TMP-TAMRA. **B**, Subcellular localization of LANA-eDHFR or eDHFR-LANA. LANA-eDHFR- or eDHFR-LANA-transfected HEK293T cells were treated with nocodazole (330 nM) for 4 h, followed by TMP-TAMRA (10 μM) with nocodazole for 1 h. DNA was stained with Hoechst 33342 to visualize chromatin distribution. Representative images of mitotic cells are shown. Scale bar, 10 μm. **C**, In-cell histone acetylation by LANA-eDHFR or eDHFR-LANA. PLIED-L52-FLAG-, LANA-eDHFR-, or eDHFR-LANA-transfected HEK293T cells were incubated with acetyl donor **2** (100 μM) and TMP-BAHA **1** (1 μM) at 37 °C for 5 h. Whole-cell extracts were immunoblotted with anti-H2BK120ac antibody or anti-H3 antibody. Representative data of two independent experiments are shown. **D**, HEK293T or HeLaS3 cells were transfected with indicated plasmids at 37 °C for 34 h. After incubation, the cell viability was measured using CellTiter-Glo 2.0 and compared to non-transfected cells. The error bars represent the range of two independent experiments.

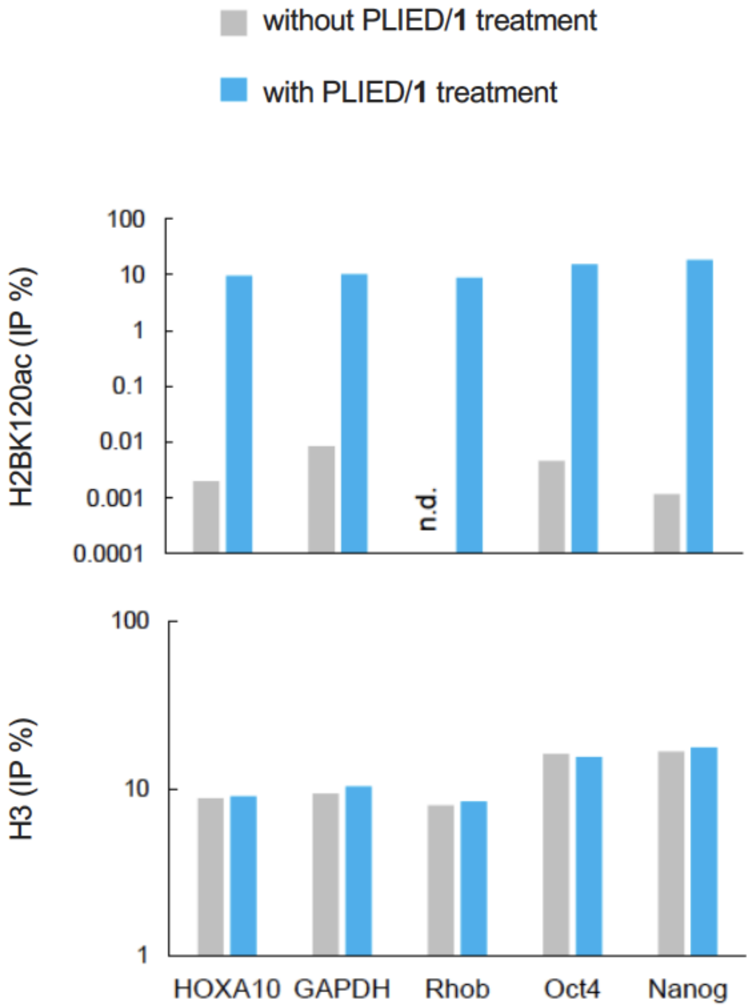

Figure S4, related to Figure 4.

Chromatin immunoprecipitation (ChIP) analysis of PLIED-L52-mediated histone acetylation. PLIED-L52-FLAG-transfected HEK293T cells were treated with TMP-BAHA **1** (5 μM) and acetyl donor **2** (200 μM) (blue bars) or **1** (5 μM) only (gray bars) at 37 °C for 10 h, and were analyzed by ChIP assays using anti-H2BK120ac antibody and anti-H3 antibody. Immunoprecipitated DNA was assessed by real-time PCR using primers specific for indicated gene locus (see also Table S8). The IP (%) were calculated from the mean values of 3 PCRs from an experiment. “n.d.” denotes “not detected”.

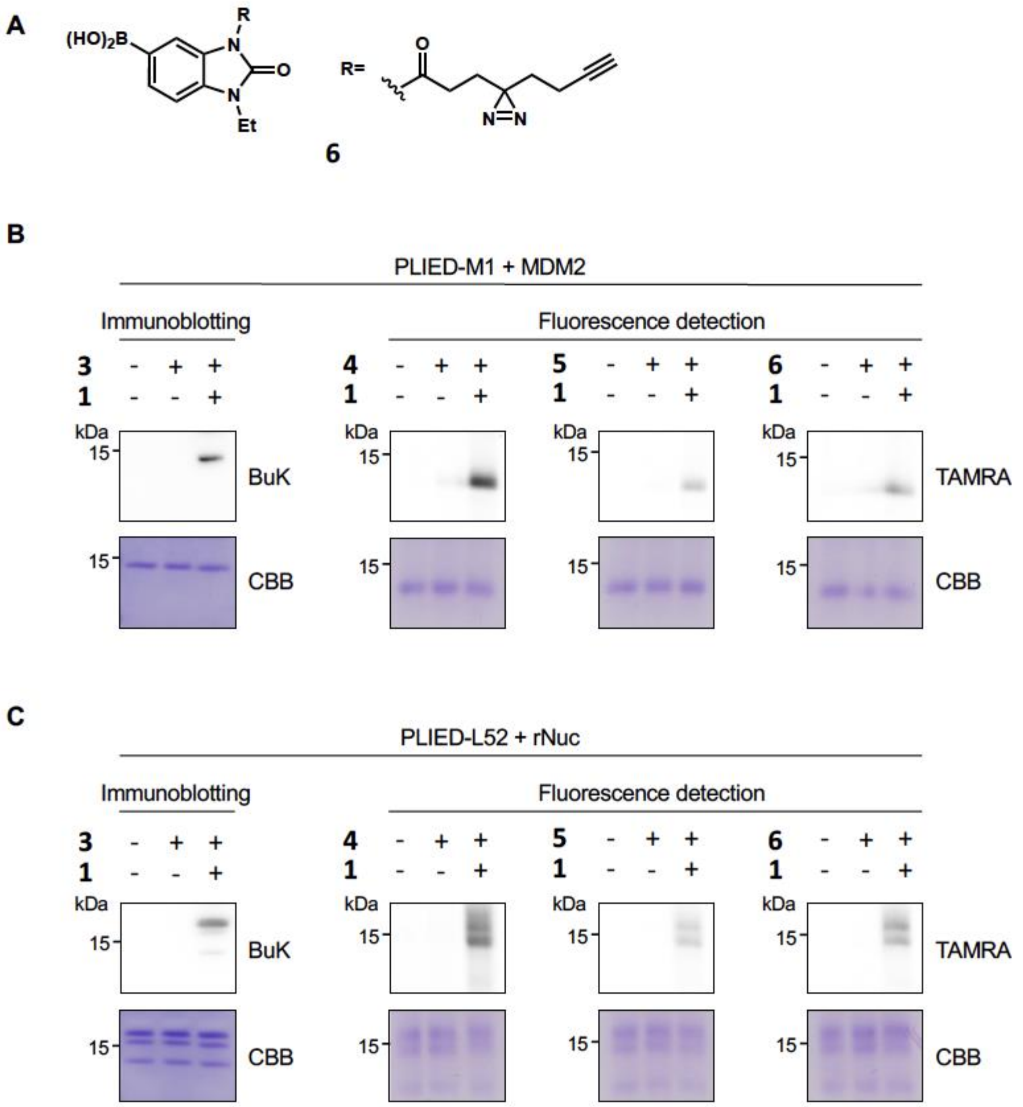

Figure S5, related to Figure 5.

A, Chemical structures of alkyne/diazirine donor **6**. **B**, Recombinant MDM2 (17–125, 1.4 μM) was incubated with PLIED-M1 in the presence or absence of TMP-BAHA **1** (10 μM) and acyl donor **3**-**6** (100 μM) at 37 °C for 5 h. **C**, Recombinant nucleosomes (0.35 μM) were incubated with PLIED-L52 protein (2 μM) in the presence or absence of TMP-BAHA **1** (5 μM) and acyl donor **3**-**6** (100 μM) at 37 ºC for 5 h. The lysine butyrylation was detected by immunoblotting using anti-butyryl lysine (BuK) antibody. To detect acylation containing azide or alkyne, acylated lysines were labelled with TAMRA-alkyne or TAMRA-azide, respectively, by Cu(I)-catalyzed azide-alkyne cycloaddition reaction. The fluorescence detection is shown (TAMRA). MDM2 and histones were visualized by CBB staining.

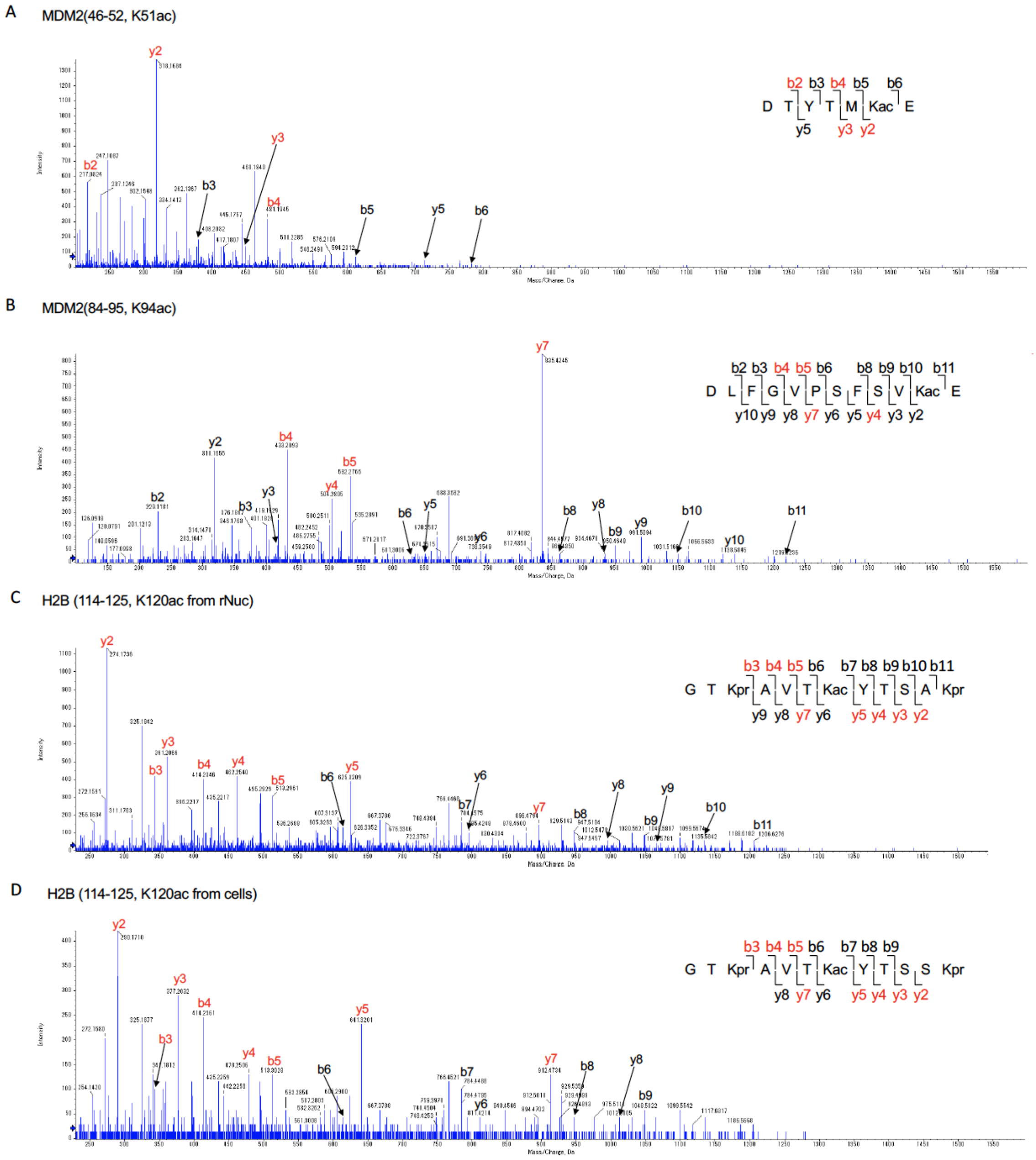

Figure S6, related to Figure 2-4.
Representative LC-MS/MS spectra for analysis of acetylation sites. MS/MS spectra of the precursor ions of acetylated peptides with m/z of 465.20 (MDM2 46-52 in Figure 2C; **A**), 683.85 (MDM2 84-95 in Figure 2H; **B**), 704.89 (H2B 114-125 from rNuc in Figure 3G; **C**), and 712.89 (H2B 114-125 from cells in Figure 4C; **D**) are shown. The b and y ions used for calculation of the percentage of lysine acetylation are colored in red.

Table S1. Plasmids for protein expression.

plasmids	expressed protein	expression system
pGEX-6P-2-hMDM2(17-125)	GST-hMDM2(17-125)	*E.Coli* BL21C+
pGEX-6P-2-eDHFR(K32R)	GST-eDHFR(K32R)	*E.Coli* BL21C+
pGEX-6P-2-eDHFR(K32R)-MBP1	GST- eDHFR(K32R)-MBP1	*E.Coli* BL21C+
pGEX-6P-2-MBP1-eDHFR(K32R)	GST- MBP1-eDHFR(K32R)	*E.Coli* BL21C+
pGEX-6P-2-PLIED-M1(K32R)	GST-PLIED-M1(K32R)	*E.Coli* BL21C+
pGEX-6P-2-PLIED-M2(K32R)	GST-PLIED-M2(K32R)	*E.Coli* BL21C+
pGEX-6P-2-PLIED-M3(K32R)	GST-PLIED-M3(K32R)	*E.Coli* BL21C+
pGEX-6P-2-LANA-eDHFR(K32R)	GST-LANA-eDHFR(K32R)	*E.Coli* BL21C+
pGEX-6P-2-eDHFR(K32R)-LANA	GST-eDHFR(K32R)-LANA	*E.Coli* BL21C+
pGEX-6P-2-PLIED-L23(K32R)	GST-PLIED-L23(K32R)	*E.Coli* BL21C+
pGEX-6P-2-PLIED-L36(K32R)	GST-PLIED-L36(K32R)	*E.Coli* BL21C+
pGEX-6P-2-PLIED-L51(K32R)	GST-PLIED-L51(K32R)	*E.Coli* BL21C+
pGEX-6P-2-PLIED-52(K32R)	GST-PLIED-L52(K32R)	*E.Coli* BL21C+
pcDNA5/TO-eDHFR(K32R)-FLAG	eDHFR(K32R)-FLAG	HEK293T
pcDNA5/TO-LANA-eDHFR(K32R)	LANA-eDHFR(K32R)	HEK293T
pcDNA5/TO-eDHFR(K32R)-LANA	eDHFR(K32R)-LANA	HEK293T
pcDNA5/TO-PLIED-L23(K32R)-FLAG	PLIED-L23(K32R)-FLAG	HEK293T
pcDNA5/TO-PLIED-L36(K32R)-FLAG	PLIED-L36(K32R)-FLAG	HEK293T
pcDNA5/TO-PLIED-L51(K32R)-FLAG	PLIED-L51(K32R)-FLAG	HEK293T
pcDNA5/TO-PLIED-L52(K32R)-FLAG	PLIED-L52(K32R)-FLAG	HEK293T, HeLaS3

Table S2. LC–MS/MS parameters for MDM2.

peptide	sequence	digestion	precuesor ion (*m/z*)[a]	fragment ions	collision energy (V)	retention time (min)
46-52	DTYTMKE	Glu-C/Asp-N	472.21 (1Pr)<br>465.20 (1Ac)	b_2, b_4, y_2, y_3	35	3.9-5.5
53-67	VLFYLGQYIMTKRLY	Glu-C/Asp-N	655.36 (1Pr)<br>650.69 (1Ac)	y_5, y_6, y_7, y_8	35	7.3-8.6
84-95	DLFGVPSFSVKE	Glu-C/Asp-N	690.86 (1Pr)<br>683.85 (1Ac)	b_4, b_5, y_4, y_7	35	7.1-8.4
96-114	HRKIYTMIYRNLVVVNQQE	Glu-C/Asp-N	820.78 (1Pr)<br>816.11 (1Ac)	y_4, y_5, y_6, y_7	35	5.3-6.5

[a] *n*Ac *m*Pr in parenthesis indicats *n* lysines acetylated out of all (*n*+*m*) lysines on the corresponding peptide.

Table S3. LC–MS/MS parameters for histone H2A.

peptide	sequence	digestion	precuesor ion (m/z)[a]	fragment ions	collision energy (V)	retention time (min)
4-11	GKQGGKAR	trypsin	457.26 (2Pr)<br>450.26 (1Pr 1Ac)<br>443.25 (2Ac)	y_4, y_5, y_6	35	2.0-3.6
12-17	AKAKTR	trypsin	393.75 (2Pr)<br>386.74 (1Pr 1Ac)<br>379.73 (2Ac)	b_2, b_3, y_3, y_4	35	1.7-3.6
36-42	KGNYSER	trypsin	455.22 (1Pr)<br>448.22 (1Ac)	y_5, y_6, y_7, y_8	35	2.7-3.4
72-77	DNKKTR	trypsin	437.24 (2Pr)<br>430.24 (1Pr 1Ac)<br>423.23 (2Ac)	b_3, y_3	35	2.8-3.6
93-99	LNKLLGR	trypsin/Glu-C	435.28 (1Pr)<br>428.27 (1Ac)	y_3, y_4, y_5, y_6	35	4.1-4.7
100-121	VTIAQGGVLPNI QAVLLPKKTE	trypsin/Glu-C	801.14 (2Pr)<br>796.47 (1Pr 1Ac)<br>791.80 (2Ac)	y_5, y_6, y_7, y_9	35	7.2-7.7
122-129	SHHKAKGK	trypsin/Glu-C	530.80 (3Pr)<br>523.79 (2Pr 1Ac)<br>516.78 (1Pr 2Ac)<br>509.78 (3Ac)	b_4, b_5, y_3, y_4<br>b_6, b_7, y_2	35	2.5-3.7

[a] *n*Ac *m*Pr in parenthesis indicats *n* lysines acetylated out of all (*n*+*m*) lysines on the corresponding peptide.

Table S4. LC–MS/MS parameters for histone H2B.

peptide	sequence	digestion	precuesor ion (*m/z*)[a]	fragment ions	collision energy (V)	retention time (min)
25-29	DGKKR	trypsin/Asp-N	358.21 (2Pr)<br>351.20 (1Pr 1Ac)<br>344.19 (2Ac)	b_3, y_2	35	3.3-3.4
34-50	KESYSIYVYKVLKQVHP	trypsin/Asp-N	750.41 (3Pr)<br>745.74 (2Pr 1Ac)<br>741.07 (1Pr 2Ac)<br>736.40 (3Ac)	b_8, y_8, y_9<br>y_5, y_6, y_7	35	7.7-7.9
51-67	DTGISSKAMGIMNSFVN	trypsin/Asp-N	914.43 (1Pr)<br>907.43 (1Ac)	b_8, b_9, b_{11}, y_6, y_8	35	7.1-7.3
80-86	LAHYNKR	trypsin	479.27 (1Pr)<br>472.26 (1Ac)	y_3, y_4, y_5, y_6	35	2.5-2.8
106-113	LAKHAVSE	trypsin/Glu-C	455.75 (1Pr)<br>448.75 (1Ac)	b_3, b_4, b_5, b_6	35	2.8-3.3
114-125 (rNuc)	GTKAVTKYTSAK	trypsin/Glu-C	711.90 (3Pr)<br>704.89 (2Pr 1Ac)<br>697.88 (1Pr 2Ac)<br>690.87 (3Ac)	b_3, b_4, b_5, y_7<br>y_2, y_3, y_4, y_5	40	4.2-5.9
114-125 (cell)	GTKAVTKYTSSK	trypsin/Glu-C	719.89 (3Pr)<br>712.89 (2Pr 1Ac)<br>705.88 (1Pr 2Ac)<br>698.87 (3Ac)	b_3, b_4, b_5, y_7<br>y_2, y_3, y_4, y_5	40	4.1-5.5

[a] *n*Ac *m*Pr in parenthesis indicats *n* lysines acetylated out of all (*n*+*m*) lysines on the corresponding peptide.

Table S5. LC–MS/MS parameters for histone H3.

peptide	sequence	digestion	precuesor ion (m/z)[a]	fragment ions	collision energy (V)	retention time (min)
3-8	TKQTAR	trypsin	380.72 (1Pr)<br>373.71 (1Ac)	y_2, y_3, y_4, y_5	35	1.6-3.0
9-17	KSTGGKAPR	trypsin	507.29 (2Pr)<br>500.28 (1Pr 1Ac)<br>493.27 (2Ac)	y_5, y_6, y_7, y_8	35	2.6-3.1
18-26	KQLATKAAR	trypsin	549.84 (2Pr)<br>542.83 (1Pr 1Ac)<br>535.82 (2Ac)	y_5, y_6, y_7, y_8	35	3.5-4.6
27-40	KSAPATGGVKKPHR	trypsin	534.64 (3Pr)<br>529.97 (2Pr 1Ac)<br>525.30 (1Pr 2Ac)<br>520.63 (3Ac)	y_5, y_6, y_7, y_8<br>y_4	35	3.4-4.1
54-63	YQKSTELLIR	trypsin	653.87 (1Pr)<br>646.86 (1Ac)	b_3, y_6, y_7, y_8	35	4.7-5.3
64-69	KLPFQR	trypsin	422.76 (1Pr)<br>415.75 (1Ac)	y_2, y_3, y_4, y_5	35	4.2-4.9
73-83	EIAQDFKTDLR	trypsin	696.36 (1Pr)<br>689.35 (1Ac)	y_5, y_6, y_7, y_8	35	4.9-6.0
117-128	VTIMPKDIQLAR	trypsin	720.92 (1Pr)<br>713.91 (1Ac)	b_3, y_8, y_9, y_{10}	35	5.1-5.8

[a] nAc mPr in parenthesis indicats n lysines acetylated out of all (n+m) lysines on the corresponding peptide.

Table S6. LC–MS/MS parameters for histone H4.

peptide	sequence	digestion	precuesor ion (m/z)[a]	fragment ions	collision energy (V)	retention time (min)
4-17	GKGGKGLGKGGAKR	trypsin	747.94 (4Pr)<br>740.93 (3Pr 1Ac)<br>733.93 (2Pr 2Ac)<br>726.92 (1Pr 3Ac)<br>719.91 (4Ac)	y_3, y_4, y_5<br>b_2, b_3, b_4<br>y_7, y_8, y_9	45	3.8-5.1
20-23	KVLR	trypsin	286.20 (1Pr)<br>279.19 (1Ac)	y_2, y_3	35	3.0-4.0
24-35	DNIQGITKPAIR	trypsin	691.39 (1Pr)<br>684.39 (1Ac)	y_6, y_7, y_8, y_9	40	4.3-4.8
41-45	GGVKR	trypsin	286.68 (1Pr)<br>279.67 (1Ac)	y_2, y_3	35	2.3-3.0
56-67	GVLKVFLENVIR	trypsin	721.94 (1Pr)<br>714.93 (1Ac)	y_6, y_7, y_8, y_9	40	7.3-7.8
68-78	DAVTYTEHAKR	trypsin	673.84 (1Pr)<br>666.83 (1Ac)	y_5, y_6, y_7, y_8	45	3.0-4.3
79-92	KTVTAMDVVYALKR	trypsin	853.98 (2Pr)<br>846.97 (1Pr 1Ac)<br>839.96 (2Ac)	y_8, y_9, y_{10}, y_{11}	40	6.4-6.8

[a] nAc mPr in parenthesis indicats n lysines acetylated out of all (n+m) lysines on the corresponding peptide.

Table S7. Antibodies for western blotting or ChIP assay[4].

antibodies	source	identifier
Anti-acetyl lysine	cell signaling technology	9441
Anti-H2BK120ac	ref. 2	N/A
Anti-butyryl lysine	PTM Biolabs	PTM-301
Anti-RNF20	Novus	NB100-2242
Anti-b-actin	sigma	A5316
Anti-H2BK120ub	cell signaling technology	5546
Anti-H3K79me2	abcam	ab3594
Anti-H3K9ac	millipore	07-352
Anti-H3K18ac	abcam	ab1191
Anti-H3	abcam	ab1791
Normal rabbit IgG	cell signaling technology	2729
Normal mouse IgG	SantaCruz	sc-2025

Table S8. Primers for real-time PCR[5].

primers	sequene (5'-3')	reference
HOXA10-fw	TGGACCAATGATGCCCTTCT	5
HOXA10-rv	CCTGATTGCCCAAGACTCGA	
GAPDH-fw	CCGGGAGAAGCTGAGTCATG	5
GAPDH-rv	TTTGCGGTGGAAATGTCCTT	
Rhob-fw	CCTGGTGGCCAACAAAAAAG	5
Rhob-rv	TCTGTGCGGACATGCTCGT	
Oct4-fw	GTGGAGGAAGCTGACAACAA	5
Oct4-rv	ATTCTCCAGGTTGCCTCTCA	
Nanog-fw	CAAAGGCAAACAACCCACTT	5
Nanog-rv	TCTGCTGGAGGCTGAGGTAT	
