# Supplementary material for: Designer Adaptor Proteins for Functional Conversion of Peptides to Small-Molecule Ligands toward In-Cell Catalytic Protein Modification
Source: ACS Cent Sci. 2023 Oct 25;9(11):2115–28. doi: 10.1021/acscentsci.3c00930 (PMC10683481; doi:10.1021/acscentsci.3c00930)
Supplement: Supplementary file 2 — oc3c00930_si_002.pdf [file oc3c00930_si_002.pdf]

oc-2023-009305.R1

Name: Peer Review Information for "Designer adaptor proteins for functional conversion of peptides to small-molecule ligands toward in-cell catalytic protein modification"

First Round of Reviewer Comments

Reviewer: 1

Comments to the Author

In this manuscript, the Kanai group build on their previous efforts to develop tools for the chemical modification of protein lysine sidechains using affinity-directed acylation catalysts. In the current installment, they have combined their previously reported methotrexate-based catalyst, TMP-BAHA, with engineered DHFR constructs containing appended or embedded peptide ligands that serve as targeting vectors for proteins of interest (POI), in the present study MDM2 and histones. Basically, the idea is that the peptide directs the DHFR, and by extension TMP-BAHA, to the POI such that proximal lysine in the complex become acylated when a boronate containing acyl donor is added to the system. This is undoubtedly a clever idea, albeit one that has a lot of moving parts. Nonetheless, the system seems to work based on a very nice series of in vitro investigations in which structural modeling and MD simulations are used to design the DHFR fusions (what they refer to as PLIED/CAT system). Indeed, there is remarkably good agreement between the sites of acetylation on the POIs found experimentally and those predicted by the modeling. Overall, the in vitro proof of concept studies are quitethorough and the results fully consistent with the design of the system.

The authors then go on to test the PLIED/CAT system in mammalian cells, focusing on chromatin where they use the well-known LANA peptide as the directing vector – the Kania group has used this peptide, which binds to the so-called acidic patch of the nucleosome, in their previous work in the general area. Consistent with the in vitro studies, they find the lysine120 on histone H2B is the major site of acetylation when the optimal PLIED system is employed. The data supporting this conclusion are solid. Less convincing are some follow up studies they perform in which they attempt to show that PLIED/CAT-mediated acetylation of H2BK120 impacts ubiquitylation at this same site as well as H3K79 methylation (which has previously been shown to be dependent on H2BK120ub). In particular, the chromatin immunoprecipitation studies (ChIP) RT-PCR data in Figure 4h are, in this reviewer's opinion, over interpreted given the errors associated with the reported measurements; the fact that H3K79me2 levels for Oct4 and Nanog are reduced to the same level as GAPDH, RhoB despite that fact that they argue H2BK120ub levels for these silenced genes are not impacted seems inconsistent and probably just reflects data quality. All this makes their argument that PLIED/CAT-mediated acetylation of H2BK120 is a better tool for probing this relationship compared to say siRNA methods less than compelling. In my

view, the manuscript would actually be improved were this part of the study simply removed. Otherwise, they will need to perform many more genomics type experiments to solidify all this which I think is not the point of this disclosure – even if they did do this, ultimately this would be of rather low impact given that the H2BK120ub-H3K79me2 axis is already so well understood. Rather, I think the study would be greatly strengthened by further development of the types of experiments in Figure 5 where other acyl units containing clickable handles are installed using the PLIED/CAT system. For example, can they get PLIED/CAT to work on other proteins in cells such as MDM2, or even in the test tube? The authors also mention that this could be used for “chromosome analysis” or incorporating crosslinkers in proteins such as histones. Perhaps this could be developed a bit more since this seems very promising.

In conclusion, I think from a protein modification perspective the study is quite innovate. However, I feel that the arguments put forward for the utility of the system in cells, particularly for the H2BK120 acylation stuff, are somewhat weak given the data currently provided.

Reviewer: 2

Comments to the Author

In this manuscript titled “Designer adaptor proteins for functional conversion of peptides to small-molecule ligands”, Fujimura et al presents a method (PLIED/CAT system) to site-specifically incorporate acyl group into proteins that can be targeted by known peptide ligands. The development of the PLIED/CAT system is an extension of the previous BAHA system developed by the authors, which used small molecule ligands to guide the lysine acylation on protein of interest. In this manuscript, the authors engineered one target protein of their BAHA system – eDHFR and incorporated different peptide ligands into the protein, the ligands can subsequently guide the catalytic lysine acylation to happen on their target protein. By predicting binding models, the specificity of the acylation can be controlled to some extent. As examples, site-specific incorporation of acetylation on MDM2 and histone H2B were demonstrated using MBP and LANA as their peptide ligands, respectively. Following the development of the PLIED/CAT system, the authors also demonstrated the application of the system in living cells. The authors showed that synthetic H2BK120 acetylation by their method can work as a protecting group at H2BK120 and thus inhibits H2BK120 ubiquitination in cells. Finally, the authors demonstrated that the system can be used to incorporate other acylations, such as butyrylation and unnatural acylation that contains azide or alkyne group. Overall, Kanai and coworkers introduce a novel method to incorporate acylations and the manuscript is well-written. I have some concerns about the results of the experiment, which I listed below:

1. The catalyst effective region (CER) is ~12Å, within which any lysine residues are modified by the acetyl group. Therefore, there is a poor labelling selectivity in the residues located within the CER. In this article, the authors utilized the PLIED-L52 system to obtain the H2BK120ub both in vitro and in living cell. However, other lysine residues in close proximity to H2B K120, e.g., H2B K116, are significantly acetylated by more than 10%, as shown in Figures 3g, h, and 4c. Therefore, although H2BK120 has the highest acetylation level, it is still important not to overlook the biological implications of the other

acetylation on neighboring sites. In the discussion, the author should comment on the unique biological impact from H2BK120ub decrease in living cells.

2. In figure 4h, left panel, the author concludes that the H2BK120ub level in heterochromatic gene (Oct4 and Nanog) was not significantly affected by PLIED-L52 acetylation. But, there is a significant increase of ub level in Nanog. In addition, in figure 4h, right panel, a significant decrease in methylation level on heterochromatic gene Oct4 and Nanog was observed. According to the author's conclusion, the methylation of Oct4 and Nanog should be unchanged because the ub levels of those genes remain unchanged. The author should repeat the Chip experiment to have high confidence results. If the results still change significantly, the author should discuss possible reasons.

3. In Supplementary Fig4, why results of the relative enrichment (H2BK120ac/H3) of the genes could support the statement of "equal level of H2BK120 acetylation"? Besides, the author should quantitatively compare changes in H2BK120ac level before and after PLIED/1 treatment. Theoretically, it should be possible to observe a significant increase in ac levels in euchromatin genes and no change in ac levels in heterochromatin genes after PLIED/1 treatment.

4. For the CHIP experiments, can the authors justify the selection of these genes in more detail? In addition, the author should further show the data treatment of all the Chip experiments.

5. The PLIED/CAT system relies on the expression of e.coli DHFR in living cells. Is there any impact on the cells when this protein is overexpressed? This should be demonstrated at two or three common cell lines.

6. Introduction of a peptide ligand into eDHFR protein might either affect the folding of eDHFR (thus affect its binding to TMP) or change the secondary structure of the peptide ligand (thus affect its binding to protein of interest), both circumstances may affect the application of PLIED/CAT system. Please comment.

7. In the MDM2 example, the authors choose to replace G51-G56 of eDHFR by the peptide ligands (page 6, line 5). However, in the histone example, the authors insert LANA between eDHFR G51 and R52 (page 7, line45). What are the considerations behind this difference?

8. The title "Designer adaptor proteins for functional conversion of peptides to small-molecule ligands" overstates the scope of the manuscript. The conversion of peptides to small molecule is limited to the use of BAHA system previously developed by the authors.

9. Please provide representative MS/MS spectrum for analysis of acylation sites.

Author's Response to Peer Review Comments:

Dear Prof:

We are grateful to you and the reviewers for carefully examining our manuscript ("**Designer adaptor proteins for functional conversion of peptides to small-molecule ligands toward in-cell catalytic protein modification**" by Akiko Fujimura, Hisashi Ishida, Tamiko Nozaki, Shuhei Terada, Yuto

Azumaya, Tadashi Ishiguro, Yugo R. Kamimura, Tomoya Kujirai, Hitoshi Kurumizaka, Hidetoshi Kono, Kenzo Yamatsugu, Shigehiro A. Kawashima, and Motomu Kanai, **Manuscript ID: oc-2023-009305)**. We are pleased that both of two reviewers have indicated that our study, if properly revised, is worthy of being published in ACS Central Science. We are now submitting a revised manuscript that has been modified to address all the concerns raised by the reviewers.

A point-by-point response to all the comments by the reviewers is attached. The major changes are:

1) We have synthesized a new acyl donor **6**, containing diazirine crosslinker and an alkyne moiety, and showed that our system can be used for incorporation of various acyl units containing clickable handles as well as a diazirine crosslinker on histones and MDM2. This revision has addressed concerns raised by reviewer #1, and has been included in new Figure S5.
2) Reviewer #1 suggested that our manuscript would be improved by removing the ChIP data in Figure 4h, since the ChIP data was not the point of our manuscript. We agree with reviewer #1's opinion and have decided to remove previous Figure 4h and related sentences from our manuscript.
3) We have changed the title to "Designer adaptor proteins for functional conversion of peptides to smallmolecule ligands toward in-cell catalytic protein modification.". This revision has addressed concerns raised by reviewer #2.

These additional data and changes have improved our manuscript and strengthened our conclusions. We do hope that you find this revised version of our manuscript to be suitable for publication in ACS Central Science.

Thank you for your time and effort in handling our manuscript.

Yours faithfully,

Motomu Kanai
Email: kanai@mol.f.u-tokyo.ac.jp
Professor
Graduate School of Pharmaceutical Sciences, The University of Tokyo

A point by point response to the reviewers' comments

Reviewer #1:

Recommendation: Reconsider after major revisions noted.

We are pleased that you have indicated that our study, if properly revised, is worthy of being published in ACS Central Science. A point-by-point response to your comments is attached below.

Comments:

In this manuscript, the Kanai group build on their previous efforts to develop tools for the chemical modification of protein lysine sidechains using affinity-directed acylation catalysts. In the current installment, they have combined their previously reported methotrexate-based catalyst, TMP-BAHA,

with engineered DHFR constructs containing appended or embedded peptide ligands that serve as targeting vectors for proteins of interest (POI), in the present study MDM2 and histones. Basically, the idea is that the peptide directs the DHFR, and by extension TMP-BAHA, to the POI such that proximal lysine in the complex become acylated when a boronate containing acyl donor is added to the system. This is undoubtedly a clever idea, albeit one that has a lot of moving parts. Nonetheless, the system seems to work based on a very nice series of in vitro investigations in which structural modeling and MD simulations are used to design the DHFR fusions (what they refer to as PLIED/CAT system). Indeed, there is remarkably good agreement between the sites of acetylation on the POIs found experimentally and those predicted by the modeling. Overall, the in vitro proof of concept studies are quitethorough and the results fully consistent with the design of the system. The authors then go on to test the PLIED/CAT system in mammalian cells, focusing on chromatin where they use the well-known LANA peptide as the directing vector – the Kania group has used this peptide, which binds to the so-called acidic patch of the nucleosome, in their previous work in the general area. Consistent with the in vitro studies, they find the lysine120 on histone H2B is the major site of acetylation when the optimal PLIED system is employed. The data supporting this conclusion are solid. Less convincing are some follow up studies they perform in which they attempt to show that PLIED/CATmediated acetylation of H2BK120 impacts ubiquitylation at this same site as well as H3K79 methylation (which has previously been shown to be dependent on H2BK120ub). In particular, the chromatin immunoprecipitation studies (ChIP) RT-PCR data in Figure 4h are, in this reviewer's opinion, over interpreted given the errors associated with the reported measurements; the fact that H3K79me2 levels for Oct4 and Nanog are reduced to the same level as GAPDH, RhoB despite that fact that they argue H2BK120ub levels for these silenced genes are not impacted seems inconsistent and probably just reflects data quality. All this makes their argument that PLIED/CAT-mediated acetylation of H2BK120 is a better tool for probing this relationship compared to say siRNA methods less than compelling. In my view, the manuscript would actually be improved were this part of the study simply removed. Otherwise, they will need to perform many more genomics type experiments to solidify all this which I think is not the point of this disclosure – even if they did do this, ultimately this would be of rather low impact given that the H2BK120ubH3K79me2 axis is already so well understood.

Thank you for carefully reading our manuscript. We appreciate for your insightful comments and completely agree with your opinions. As you suggested, we decided to remove ChIP-qPCR data in the previous Figure 4h and related sentences in the text.

Rather, I think the study would be greatly strengthened by further development of the types of experiments in Figure 5 where other acyl units containing clickable handles are installed using the PLIED/CAT system. For example, can they get PLIED/CAT to work on other proteins in cells such as MDM2, or even in the test tube? The authors also mention that this could be used for "chromosome analysis" or incorporating crosslinkers in proteins such as histones. Perhaps this could be developed a bit more since this seems very promising.

Thanks for your great suggestion. As you mentioned, we examined the PLIED/CAT system for introduction of a clickable azide handle on MDM2. Furthermore, we synthesized new acyl donor **6,** containing diazirine crosslinker and an alkyne moiety. As expected, our system can be used for incorporation of various acyl units containing clickable handles as well as a diazirine crosslinker on histones and MDM2 (see new Figure S5).

In conclusion, I think from a protein modification perspective the study is quite innovate. However, I feel that the arguments put forward for the utility of the system in cells, particularly for the H2BK120 acylation stuff, are somewhat weak given the data currently provided.

Thanks to your truly valuable comments, our revised manuscript is now much improved.

Reviewer #2:

Recommendation: Reconsider after major revisions noted.

Comments:

In this manuscript titled “Designer adaptor proteins for functional conversion of peptides to small-molecule ligands”, Fujimura et al presents a method (PLIED/CAT system) to sitespecifically incorporate acyl group into proteins that can be targeted by known peptide ligands. The development of the PLIED/CAT system is an extension of the previous BAHA system developed by the authors, which used small molecule ligands to guide the lysine acylation on protein of interest. In this manuscript, the authors engineered one target protein of their BAHA system – eDHFR and incorporated different peptide ligands into the protein, the ligands can subsequently guide the catalytic lysine acylation to happen on their target protein. By predicting binding models, the specificity of the acylation can be controlled to some extent. As examples, site-specific incorporation of acetylation on MDM2 and histone H2B were demonstrated using MBP and LANA as their peptide ligands, respectively. Following the development of the PLIED/CAT system, the authors also demonstrated the application of the system in living cells. The authors showed that synthetic H2BK120 acetylation by their method can work as a protecting group at H2BK120 and thus inhibits H2BK120 ubiquitination in cells. Finally, the authors demonstrated that the system can be used to incorporate other acylations, such as butyrylation and unnatural acylation that contains azide or alkyne group. Overall, Kanai and coworkers introduce a novel method to incorporate acylations and the manuscript is well-written.

I have some concerns about the results of the experiment, which I listed below:

Thank you for carefully reading our manuscript. We are pleased that you have indicated that our study, if properly revised, is worthy of being published in ACS Central Science. A point-by-point response to your comments is attached below.

1. The catalyst effective region (CER) is ~12Å, within which any lysine residues are modified by the acetyl group. Therefore, there is a poor labelling selectivity in the residues located within the CER. In this article, the authors utilized the PLIED-L52 system to obtain the H2BK120ub both in vitro and in living cell. However, other lysine residues in close proximity to H2B K120, e.g., H2B K116, are significantly acetylated by more than 10%, as shown in Figures 3g, h, and 4c. Therefore, although H2BK120 has the highest acetylation level, it is still important not to overlook the biological implications of the other acetylation on neighboring sites. In the discussion, the author should comment on the unique biological impact from H2BK120ub decrease in living cells.

We appreciate your insightful comments. As you suggested, we have mentioned minor but still significant acetylation on neighboring sites, in the Discussion part (page 12, lane 2325) as follows; "*It should be noted, however, that other lysine residues proximate to H2BK120, such as H2BK116, were also acetylated by the PLIED-L52/**1** system, which may contribute to H2BK120ub inhibition.*").

2. In figure 4h, left panel, the author concludes that the H2BK120ub level in heterochromatic gene (Oct4 and Nanog) was not significantly affected by PLIED-L52 acetylation. But, there is a significant increase of ub level in Nanog. In addition, in figure 4h, right panel, a significant decrease in methylation level on heterochromatic gene Oct4 and Nanog was observed. According to the author's conclusion, the methylation of Oct4 and Nanog should be unchanged because the ub levels of those genes remain unchanged. The author should repeat the Chip experiment to have high confidence results. If the results still change significantly, the author should discuss possible reasons. In the previous figure 4h, we divided IP (%) of H2BK120ub (or H3K79me2) by that of H3 and showed the average +/- SD value of three independent experiments. However, thanks to your comments, we realized that this calculation method caused increased variability of the data. To make the data treatment simpler, we here showed IP (%) in each experiment as well as the average and SD of three experiments in **Figure R1** (a-d for H2BK120ub, e-h for H3K79me2) shown below.

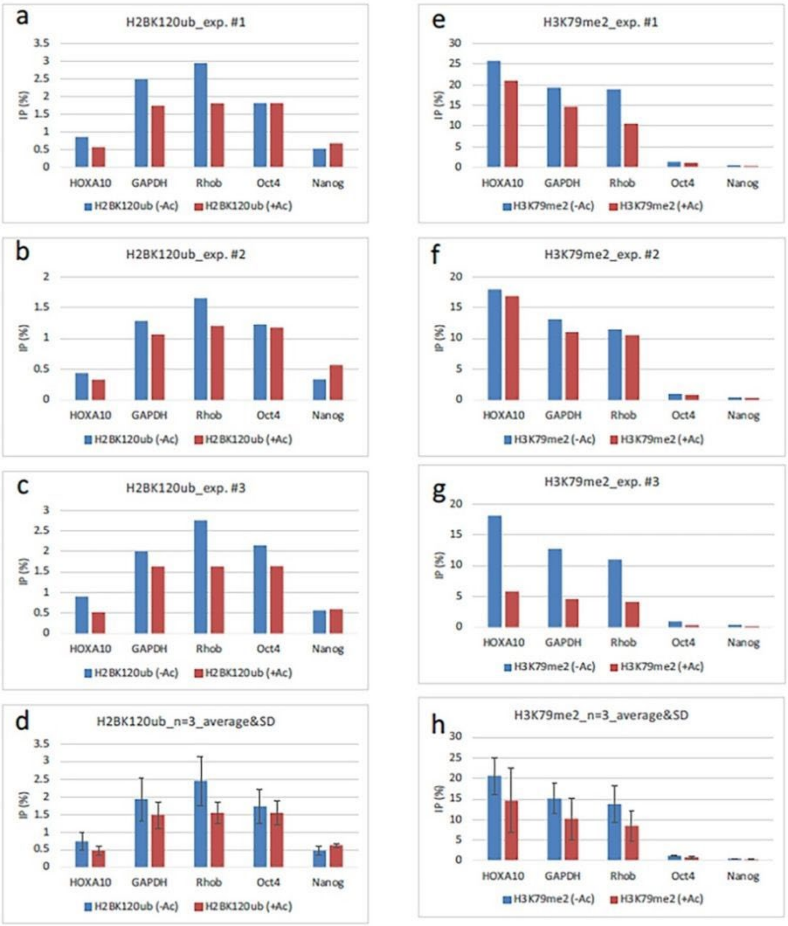

Figure. R1 The original data of ChIP analyses for H2BK120ub and H3K79me2

The results were mostly reproduced among three independent experiments, indicating high reliability of these data. The level of H2BK120ub or H3K79me2 in the HOXA10, GAPDH, or Rhob region was reduced by PLIED-L52 acetylation in all cases, so our conclusion in the original submission [the H2BK120ub or the H3K79me2 level in euchromatic gene (HOXA10, GAPDH, Rhob) was notably reduced by PLIED-L52 acetylation] is valid. However, we found that our previous description for heterochromatic regions were not appropriate, as you pointed out. The H2BK120ub level in Oct4 region was comparable in two experiments, and slightly reduced in one experiment. The H2BK120ub level in Nanog region was increased in two experiments, and comparable in one experiment. Therefore, our previous description "*the H2BK120ub level in heterochromatic gene (Oct4 and Nanog) was not significantly affected by PLIED-L52 acetylation*" might be incorrect. Rather, we have revised the sentence as follows; "*the H2BK120ub level in heterochromatic gene (Oct4 and Nanog) was not significantly* ***reduced*** *by PLIED-L52 acetylation*". The level of H3K79me2 in the heterochromatic genes (Oct4 and Nanog) was significantly lower than euchromatic regions, which is consistent with the notion that H3K79me2 is an active transcription marker. Therefore, it is difficult to precisely evaluate changes in H3K79me2 level in the heterochromatic genes. Thus, we have revised our description as follows; "*the H3K79me2 level in heterochromatic gene (Oct4 and Nanog)* ***remained low*** *after PLIED-L52 acetylation.".*

Meanwhile, reviewer #1 also commented about the ChIP data and suggested that our manuscript would be improved by removing the ChIP data in previous Fig. 4h, since the ChIP data was not the point of our manuscript (*"In my view, the manuscript would actually be improved were this part (i.e. ChIP data in Fig. 4h) of the study simply removed. Otherwise, they will need to perform many more genomics type experiments to solidify all this which I think is not the point of this disclosure – even if they did do this, ultimately this would be of rather low impact given that the H2BK120ub-H3K79me2 axis is already so well understood."*). We agree with reviewer 1's opinion and have decided to remove previous Fig. 4h and related sentences from our manuscript. We would appreciate if you kindly agree with this revision.

3. In Supplementary Fig4, why results of the relative enrichment (H2BK120ac/H3) of the genes could support the statement of "equal level of H2BK120 acetylation"? Besides, the author should quantitatively compare changes in H2BK120ac level before and after PLIED/1 treatment. Theoretically, it should be possible to observe a significant increase in ac levels in euchromatin genes and no change in ac levels in heterochromatin genes after PLIED/1 treatment.

We apologize that the label was not easy to understand, although the graph in the original Supplementary Figure 4 included H2BK120ac levels with and without PLIED/**1** treatment. To prevent misunderstanding, we have made the following four changes; 1) we have changed the label in new Figure S4 as "with PLIED/**1** treatment" and "without PLIED/1 treatment". 2) The result of H2BK120ac and H3 have been separated. 3) We have added "n.d." for the data of not detected. 4) We have changed the y-axis to log value. Based on the data, we concluded that H2BK120ac level was almost comparable between euchromatin and heterochromatin regions.

4. For the CHIP experiments, can the authors justify the selection of these genes in more detail? In addition, the author should further show the data treatment of all the Chip experiments.

Thank you for the comment. We selected HOXA10 as a H2BK120ub-positive region according to the previous report by *Shema-Yaacoby, E. et al.* in Cell Rep **2013**. GAPDH/Rhob and Oct4/Nanog were selected as they are well-known representative euchromatin and conditional heterochromatin regions, respectively. For these regions, we used the primer sets that were previously reported by David, Y. *et al.* in Nat Chem **2015**. Regarding the data treatment, we previously divided IP (%) of histone modification (H2BL120ub, H3K79me2, H2BK120ac) by that of H3, which caused increased variability of the data. To make the data treatment simpler, we now showed IP (%) of histone modification and H3 separately (new Figure S4 and Fig. R1).

5. The PLIED/CAT system relies on the expression of e.coli DHFR in living cells. Is there any impact on the cells when this protein is overexpressed? This should be demonstrated at two or three common cell lines.

We checked if the overexpression of *E.coli* DHFR affects cell viability in HEK293T and HeLa S3 cells, which are commonly used cell lines. Please see new Figure S3D. The data showed that the overexpression of eDHFR (34 hours after transfection: the same timescale as in-cell histone acetylation experiments in Figure 4F, 4G) did not affect viability in both cell lines. As controls, we used an empty

vector (vehicle) as well as the EGFP plasmid. We have added the following sentence to lane 7-8 in page 10; *The*

overexpression of eDHFR within this timescale did not affect cell viability in HEK293T and HeLa S3 cells (Figure S3D).

6. Introduction of a peptide ligand into eDHFR protein might either affect the folding of eDHFR (thus affect its binding to TMP) or change the secondary structure of the peptide ligand (thus affect its binding to protein of interest), both circumstances may affect the application of PLIED/CAT system. Please comment.

Thank you for the insightful comment. Based on EMSA assay shown in Figure 3D and Figure S2D, LANA-eDHFR, eDHFR-LANA, or PLIED-L23 has weak or no affinity to nucleosomes, suggesting that the position of a peptide ligand introduction affects the secondary structure of the peptide ligand. In addition, as shown in Figure S2B, PLIED-L36 was less soluble than other PLIEDs, suggesting that LANA insertion to position 36 affected the folding of eDHFR. PLIED-L51 and PLIED-L52 were soluble and significantly bound to nucleosomes. Therefore, the selection of a proper position for peptide ligand introduction is important to construct PLIED/CAT system. We have added the following sentence to lane 16-18 in page 8; *Therefore, the insertion of LANA to an appropriate position of eDHFR is important to construct the functional PLIED/CAT system bearing properly folded eDHFR and a peptide ligand.*

7. In the MDM2 example, the authors choose to replace G51-G56 of eDHFR by the peptide ligands (page 6, line 5). However, in the histone example, the authors insert LANA between eDHFR G51 and R52 (page 7, line45). What are the considerations behind this difference?

In the MDM2 example, the binding structures of MDM2 and MBPs (Figure 2D) showed that the binding motif of MBPs is α-helix, whose length is approximately 14.4–16.1 Å; 16.1 Å for MBP1, 14.5 Å for MBP2, and 14.4 Å for MBP3. Therefore, we replaced G51-G56 of eDHFR, whose length is approximately 15.4 Å, by the peptide ligands. In the histone example, LANA was inserted between G51 and R52 of eDHFR, since LANA has a hairpinlike structure (Figure 3A) and the distance between its N and C termini is approximately

5.6 Å.

We have included these considerations at lane 3-4 in page 6 for MDM2 and lane 28-29 in page 7 for histones in the main text.

8. The title “Designer adaptor proteins for functional conversion of peptides to smallmolecule ligands” overstates the scope of the manuscript. The conversion of peptides to small molecule is limited to the use of BAHA system previously developed by the authors. Thank you for the suggestion. We have changed the title to “Designer adaptor proteins for functional conversion of peptides to small-molecule ligands toward in-cell catalytic protein modification”.

9. Please provide representative MS/MS spectrum for analysis of acylation sites.We provided representative MS/MS spectrum for acetylation sites analysis in Figure 2C, 2H, 3G, and 4C. Please see new Fig S6.
